# Sequence Comparison of Vaginolysin from Different *Gardnerella* Species

**DOI:** 10.3390/pathogens10020086

**Published:** 2021-01-20

**Authors:** Erin M. Garcia, Myrna G. Serrano, Laahirie Edupuganti, David J. Edwards, Gregory A. Buck, Kimberly K. Jefferson

**Affiliations:** 1 Department of Microbiology and Immunology, Virginia Commonwealth University, Richmond, VA 23298, USA; egarcia26@wisc.edu (E.M.G.); myrna.serrano@vcuhealth.org (M.G.S.); Laahirie.edupuganti@vcuhealth.org (L.E.); gregory.buck@vcuhealth.org (G.A.B.); 2 Center for Microbiome Engineering and Data Analysis, Virginia Commonwealth University, Richmond, VA 23298, USA; dedwards7@vcu.edu; 3 Department of Statistical Sciences and Operations Research, Virginia Commonwealth University, Richmond, VA 23298, USA; 4 Department of Computer Science, College of Engineering, Virginia Commonwealth University, Richmond, VA 23298, USA

**Keywords:** *Gardnerella*, vaginal microbiota, bacterial vaginosis, virulence factors, vaginolysin

## Abstract

*Gardnerella vaginalis* has recently been split into 13 distinct species. In this study, we tested the hypotheses that species-specific variations in the vaginolysin (VLY) amino acid sequence could influence the interaction between the toxin and vaginal epithelial cells and that VLY variation may be one factor that distinguishes less virulent or commensal strains from more virulent strains. This was assessed by bioinformatic analyses of publicly available *Gardnerella* spp. sequences and quantification of cytotoxicity and cytokine production from purified, recombinantly produced versions of VLY. After identifying conserved differences that could distinguish distinct VLY types, we analyzed metagenomic data from a cohort of female subjects from the Vaginal Human Microbiome Project to investigate whether these different VLY types exhibited any significant associations with symptoms or *Gardnerella* spp.-relative abundance in vaginal swab samples. While Type 1 VLY was most prevalent among the subjects and may be associated with increased reports of symptoms, subjects with Type 2 VLY dominant profiles exhibited increased relative *Gardnerella* spp. abundance. Our findings suggest that amino acid differences alter the interaction of VLY with vaginal keratinocytes, which may potentiate differences in bacterial vaginosis (BV) immunopathology in vivo.

## 1. Introduction

Whole-genome sequencing and phylogenetic analysis of multiple strains have resulted in the recent taxonomic re-distribution of *Gardnerella vaginalis*, initially described in the late 1950s [1], into 13 distinct species [2]. Vaginolysin (VLY), which is produced by most *Gardnerella* spp., is a cholesterol-dependent cytolysin (CDC) that may play a role as a virulence factor [3,4]. Previous studies suggest that in vitro expression and secretion of VLY may vary among strains. Specifically, one report found that strains isolated from subjects with clinically defined bacterial vaginosis (BV) expressed two-fold more *vly* compared to strains isolated from those without clinically defined BV [5]. Two additional studies found significant differences in the level of VLY secreted from different *Gardnerella* strains, but these differences did not correlate with the genotype [6,7]. Like other CDCs, VLY requires adequate membrane cholesterol levels for oligomerization and subsequent pore formation [3,8]. However, VLY also exhibits a requirement for the human complement receptor, CD59, in that the amount of toxin needed to elicit the same amount of cytotoxicity on cells lacking human CD59 is increased by 1000-fold [8]. Like other CD59-binding CDCs, VLY has a proline residue instead of a tryptophan in its C-terminal undecapeptide domain, a motif conserved among CDCs that is involved with membrane interaction [9]. A region proximal to the undecapeptide motif was implicated in CD59 binding in the closely related intermedilysin (ILY) [10], but crystallographic analysis of VLY and ILY implicated different residues in CD59 binding [11]. VLY is toxic to a variety of cell types, including erythrocytes, epithelial cells, and neutrophils [3,4,12]. Interestingly, however, we recently reported that the apical face of a three-dimensional tissue model of human vaginal epithelium is relatively resistant to VLY, and our data also showed that there was reduced expression of CD59 on the apical side [13]. This finding seems to contradict in vivo evidence suggesting that *Gardnerella* spp. and VLY are pro-inflammatory and contribute to vaginal tissue damage in BV [14,15,16,17,18]. Multiple reports have suggested that both commensal and BV-associated strains of *Gardnerella* exist, and a recent study using high-resolution bioinformatics to enable taxonomic assignment to the subspecies level found that the association of the *Gardnerella* genus with preterm birth is attributable to a singular subspecies clade [19,20,21,22,23].

In an effort to reconcile the apparent contradiction between in vivo data that VLY elicits damage and our in vitro findings that VLY largely spares the lumenal side of the vaginal epithelium, we tested the hypotheses that species-specific variations in the VLY amino acid sequence could influence the interaction between the toxin and vaginal epithelial cells and that VLY variation may be one factor that distinguishes less virulent or commensal strains from more virulent strains. While studies have noted the high amino acid identity shared by VLYs from diverse *Gardnerella* strains (90–99%), there has yet to be a more comprehensive analysis of the level of VLY conservation among the now 91 publicly available *Gardnerella* genomes [6,19]. However, in a more recent analysis of 32 VLY amino acid sequences, one study reported a valine-to-alanine substitution variant within the undecapeptide region [24]. Comparisons among the VLYs of the recently proposed 13 distinct *Gardnerella* genomic species [2], however, have yet to be made. To determine whether VLY amino acid differences among the newly proposed species have functional consequences that may affect virulence, we analyzed VLY amino acid conservation among the publicly available *Gardnerella* genome sequences. After identifying conserved differences that could distinguish distinct VLY types, we produced recombinant toxins to test whether two distinct types affected vaginal epithelial cells differently, focusing on cytotoxicity and cytokine production. Finally, using metagenomic data from a cohort of female subjects from the Vaginal Human Microbiome Project [25] at Virginia Commonwealth University, we investigated whether these different VLY types exhibited any significant associations with symptoms reported by the subjects or the relative abundance of *Gardnerella* spp. in vaginal swab samples [26]. Our findings suggest that amino acid differences alter the interaction of VLY with vaginal keratinocytes, which may potentiate differences in BV immunopathology in vivo. These differences may determine whether or not a productive immune response is mounted and may explain why some but not all people with BV-like vaginal microbiome profiles experience symptoms.

## 2. Results

### 2.1. Amino Acid Sequence Differences Distinguish Five Vaginolysin Types

**Figure 1:**
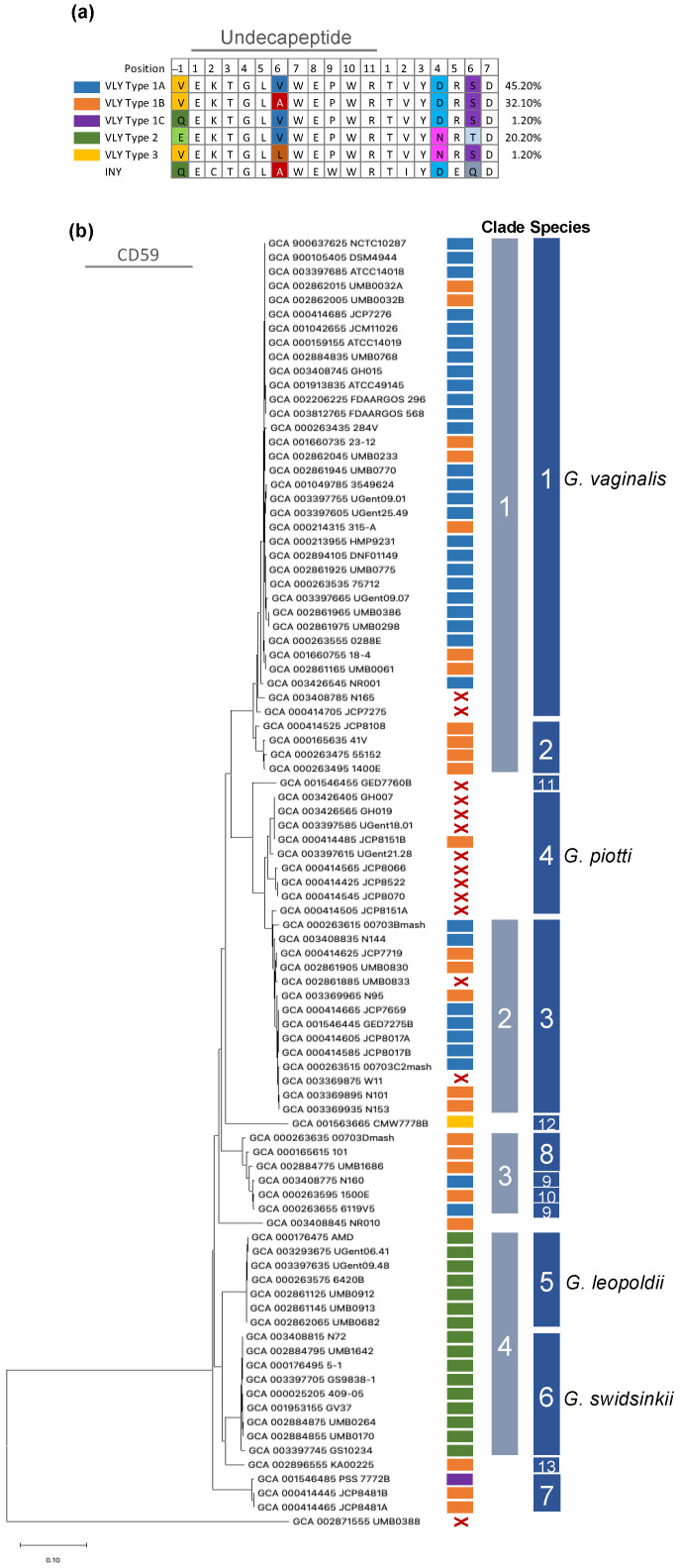
Five VLY types are distinguishable among *Gardnerella* strains. (**a**) VLY-type classification based on undecapeptide and CD59 region sequences of 94 *Gardnerella* spp. strains. Percentage values are the number of strains with a given VLY type out of all 94 strains examined. INY is the undecapeptide region from the *Lactobacillus iners* toxin, inerolysin. (**b**) Distribution of VLY types amongst *Gardnerella* genomic species. Neighbor-joining phylogenetic tree of full length cpn60 nucleotide sequences (1629 bp) of 91 *Gardnerella* spp. available genomes from GenBank). VLY types are color-coded and shown in panel (**a**). A red “X” indicates that *vly* is absent. Clade distribution as described by Ahmed et al. [29] and species distribution as described in Vaneechoutte et al. [2].

Alignment of 91 publicly available VLY sequences showed that the pairwise percent identities ranged from 90.48–100.0% with an average of 96.97% (Supplementary Figure S1). Transmembrane domains and residues that were identified to participate in CD59 binding by X-ray crystallography [11] exhibited 100% identity between all VLY sequences. Domain 4, containing the undecapeptide, proximal CD59 region, and loop regions conserved in CDCs [10,27], was highly conserved, but contained a limited number of amino acid variants (Supplementary Figure S2). Alignment of the undecapeptide and proximal CD59 regions identified five distinct vaginolysin types (Figure 1a). The undecapeptide and CD59 regions from inerolysin, a CDC produced by *Lactobacillus iners* [28], is shown for comparison. The VLY types were first distinguished by the sequence differences in the predicted CD59 region and then further sub-grouped based on sequence differences in the undecapeptide region. Type 1 VLY contained aspartate and serine in positions 4 and 6 of the CD59 region, respectively. Type 1A was characterized by valines at positions −1 and 6 of the undecapeptide region, while Type 1B had valine and alanine, and Type 1C had glutamine and valine at these positions. The CD59 region of Type 2 VLY contained asparagine and threonine at positions 4 and 6. The undecapeptide region of this type was characterized by glutamate at position −1 and valine at position 6. As there was no variability in this region, Type 2 was not further sub-classified. Finally, Type 3 VLY was characterized by asparagine and serine within the CD59 region and valine and leucine at positions −1 and 6 of the undecapeptide region. Types 1A and 1B were the most common, comprising 37.4% and 27.5% of the analyzed strains, respectively. The other types were less common: 17.6% were Type 2 and Types 1C, and three were each only represented by a single strain. In sum, this survey of VLY amino acid sequences from *Gardnerella* spp. isolates reveals heterogeneity within the regions of the toxin known to interact with host cells. 

To determine whether these different versions of the toxin are encoded by specific *Gardnerella* species, we phylogenetically mapped each available strain using *cpn60* gene sequences, grouped them into one of the 4 clades [29] and 13 proposed *Gardnerella* genomic species [2], and compared them by VLY type (Figure 1b). Type 1 VLY dominated in species 1 (*G. vaginalis*), 2, 3, 7, 8, 9, 10, and 13, whereas Type 2 was detected exclusively in species 5 (*Gardnerella leopoldii*) and 6 (*Gardnerella swidsinskii*), and Type 3 was only found in species 12. Species 4 (*Gardnerella piotii*) had a single representative strain with the Type 1 VLY, but the gene was absent in the remaining eight strains in this group, and it was absent in the single representative strain from species 11. A few other strains from species 1–3 also lacked VLY. Thus, it appears that different *Gardnerella* species tend to encode a particular VLY type, and some species tend to lack the *vly* gene altogether.

### 2.2. Cytokine Secretion Profiles of VK2 Cells Challenged with Type 1A and Type 2 VLY Are Similar

While there appeared to be five distinct VLY types based on sequence differences, we suspected that some differences had a greater potential to alter protein function than others. For example, the amino acids distinguishing Type 1A and 1B toxins are of the same amino acid class (nonpolar aliphatic), and we therefore anticipated that they would be phenotypically similar. Type 2 VLY, on the other hand, is distinguished from Type 1 VLY by the change of a polar, uncharged asparagine residue in the CD59 region to a negatively charged aspartate residue. It also has a negatively charged glutamate residue upstream of the undecapeptide region that is replaced with a nonpolar, aliphatic valine in Type 1A and Type 1B VLY. While Type 1C and Type 3 toxins exhibited sequence differences that could potentially impart functional diversity, their rarity among available *Gardnerella* strains prompted us to limit our in vitro analyses to Type 1A and Type 2 toxins. We hypothesized that differences in these proteins could result in differential interactions with vaginal epithelial cells and tested this hypothesis by quantifying their cytokine responses and cytotoxic potential. We cloned the *vly* genes from strains harboring Type 1A (*G. vaginalis* ATCC 14018) or Type 2 (*G. leopoldii* AMD) into an expression vector, expressed the proteins in *E. coli*, and purified them. Purified recombinant VLY (rVLY) was quantified by Bradford assay and assayed for purity by SDS PAGE (Supplementary Figure S3). 

**Figure 2:**
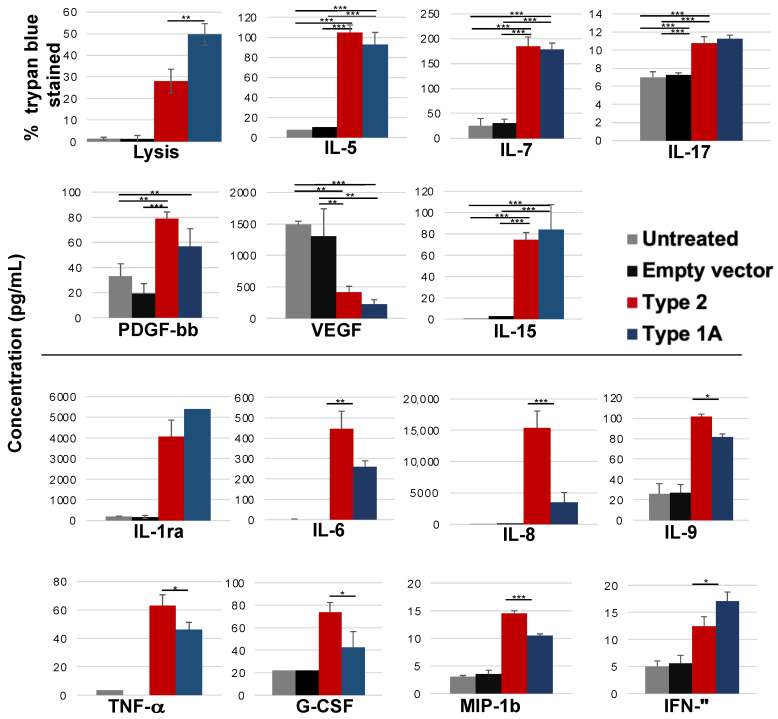
Effects of VLY types 1A and 2 on cytokine secretion. VK2 monolayers cultured in 24-well plates were treated with 1 μg VLY/mL for 19 h. Samples of spent media from the cells were then analyzed by a multiplex cytokine bead array. For cytokines secreted at levels ≥10 pg/mL, mean cytokine concentrations (pg/mL) exhibiting significantly different concentrations between untreated and VLY treated cells (top) or between type 2 and type 1A VLY (bottom) are shown. Error bars represent standard deviations. * = *p* ≤ 0.05, ** ≤ 0.01, *** ≤ 0.001 (*n* ≥ 3). Protein purified from *Escherichia coli* containing the empty expression vector was included as a control for endotoxin or other contaminants.

We assessed levels of cytokines secreted by VK2 cells into the media after 19 h of incubation with Type 1A or Type2 rVLY (Figure 2). After incubation, cells treated with Type 1A exhibited approximately 50% lysis, whereas cells treated with Type 2 exhibited approximately 30% lysis. For cytokines secreted at levels above 10 pg/mL, those that differed from controls by ≥2-fold following rVLY exposure included IL-5, IL-7, PDGF-bb, VEGF, IL-1ra, IL-6, IL-8, IL-9, IL-15, TNF-α, GCSF, MIP-1b, and IFN-γ. VEGF was the only cytokine that exhibited a lower concentration following rVLY exposure (6.7-fold). Concentrations of many cytokines were statistically different (*p* < 0.05) depending on the rVLY type added to the VK2 cells, but only IL-8 exhibited a difference ≥2-fold (Figure 2, bottom). VK2 cells challenged with Type 1A rVLY secreted 4.4-fold less IL-8 than cells challenged with Type 2 rVLY. Thus, both rVLY types elicited similar cytokine secretion profiles from VK2 cell monolayers, but there were some differences in the levels of cytokines secreted in response to treatment with either rVLY type. As the two toxins caused significantly different levels of cell permeabilization, these differences in cytokine levels could be due to differences in lysis.

### 2.3. Type 1A rVLY Exhibits Greater Cytotoxicity on Cells with Less Membrane CD59 

**Figure 3:**
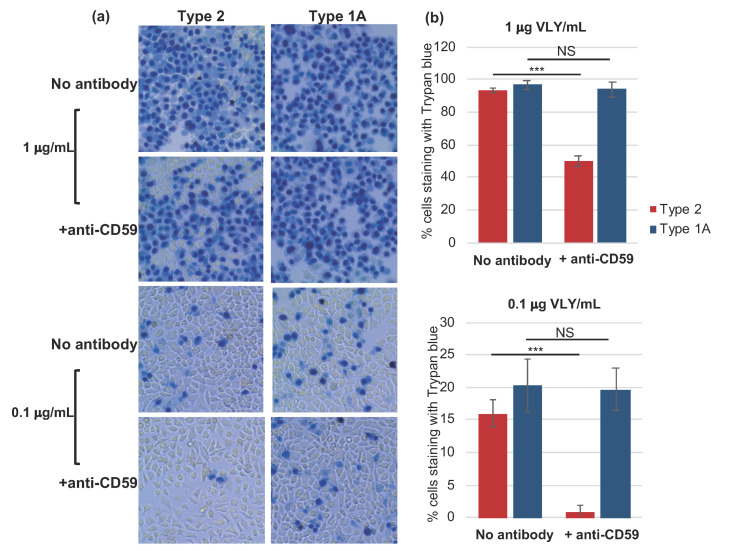
Anti-CD59 pre-treatment has a greater impact on Type 2 rVLY cytotoxicity. VK2 monolayers (4 days post-seeding) with and without pretreatment (0.2 mg/mL anti-CD59 antibody for one hour) were challenged with 1 μg or 0.1 μg VLY/mL, for 4 h. Cells were then rinsed, stained with trypan blue, and imaged. (**a**) Images of trypan-blue-stained cells. (**b**) Percentage of cells out of 1000 counted that stained with trypan blue. Assays were performed in technical and biologic triplicate and error bars represent standard deviations. NS = not significant; ***, *p* ≤ 0.001 (*n* ≥ 3).

To quantify and compare cytotoxicity, we added 1 or 0.1 µg/mL of Type 1A or Type 2 rVLY to VK2 cell monolayers and stained cells with trypan blue after 4 h of rVLY exposure. The percentage of VK2 cells that stained with trypan blue did not differ significantly between the two treatment groups (Figure 3). However, when we pre-treated cells with anti-CD59 antibody for one hour prior to rVLY challenge, we observed that the reduction in cytotoxicity was significantly greater in Type 2 rVLY-challenged cells at both toxin concentrations (Figure 3b), suggesting that rVLY Type 1A may have a higher affinity for, or depend less upon, the CD59 receptor. 

### 2.4. VaHMP Study Participants Are Simultaneously Colonized by Several Gardnerella Species

**Figure 4:**
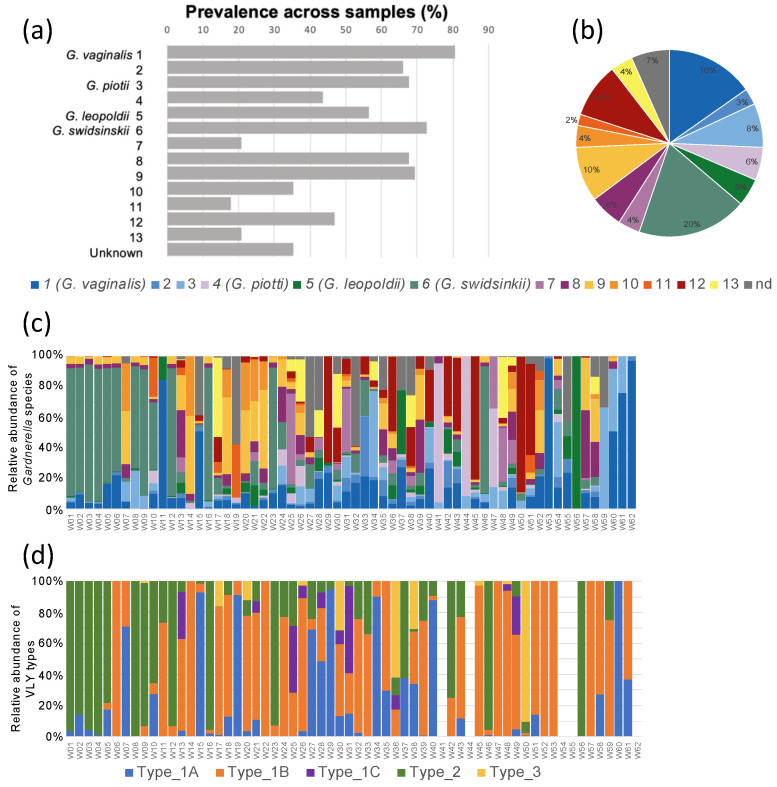
BV-positive subjects are simultaneously colonized by multiple *Gardnerella* spp. cpn60 reads were used to distinguish each *Gardnerella* genomic species. Each species is numbered according to the scheme reported in Vaneechoutte et al. 2019, and, where possible, labeled with a species designation; nd = not determined. (**a**) Percentage of participants in the cohort who were colonized by each of the 13 *Gardnerella* species. Presence of *Gardnerella* species is defined as whether the number of reads mapped to the reference is 10 or higher. “Unknown”, *Gardnerella* strain not yet classified. (**b**) Mean relative abundance of each genomic species across all samples. (**c**) Abundance of each *Gardnerella* species relative to the total *Gardnerella* population within each sample. Each bar represents one sample. Legend as indicated in panel (**b**). (**d**) Abundance of each VLY type relative to all *vly* reads (based on whole metagenome sequencing data) in each sample.

To investigate whether *Gardnerella* species identity and/or VLY type were associated with differences in proportional abundance within the vaginal microbiome or with differences in symptom presentation, we analyzed metagenomic data from a cohort of 62 female subjects from the Vaginal Human Microbiome Project [25] who had a positive diagnosis for BV and were vaginally colonized by *Gardnerella* (Supplementary Figure S4a). Since each functional VLY type is restricted to select *Gardnerella* species, we began our analyses by investigating the distribution of each *Gardnerella* species within our cohort, using *cpn60* universal target sequences to resolve distinct species (Supplementary Figure S5b) [29]. Seven of the 13 defined species were present (at least 10 reads) in at least half of the samples. These included *G. vaginalis*, *G. swidsinskii*, *G. piotii*, and *G. leopoldii* in addition to species 2, 8, and 9 (Figure 4a). *G. swidsinskii* (20.0%), *G. vaginalis* (15.58%), species 12 (9.91%), and species 9 (9.34%) were the most abundant as measured by mean abundance relative to all *Gardnerella* species across all samples (Figure 4b). Interestingly, a majority of our participants were simultaneously colonized by multiple *Gardnerella* species (Figure 4c). Most subjects (90.2%) had three or more species present in their samples, and the average number of species present per participant was nine. The abundances of each VLY type relative to all *vly* reads within each sample are shown in Figure 4d. Genomes were assembled from the metagenomic data to compare VLY amino acid sequences in our dataset to those that are publicly available. We did not detect any additional amino acid variants beyond those described above.

### 2.5. Gardnerella with Type 2 Vly Gene Reach Higher Proportional Abundance 

**Figure 5:**
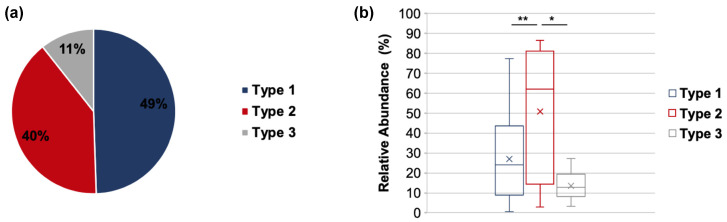
Relative abundance of different VLY Types. (**a**) The percent of each VLY type detected within the total *vly* reads for the entire cohort. Type 1 includes 1A, 1B, and 1C. (**b**) Median relative *Gardnerella* abundance in each VLY type group. “x” marks the mean relative abundance. * = *p* ≤ 0.05, ** ≤ 0.01.

We next assessed the distribution of VLY Types 1, 2, and 3 among the cohort as a whole. We did this first by calculating the number of reads of each type across all samples and dividing by the total number of all VLY type reads from all samples. Type 1 VLY represented 49.5% of the total *vly* reads, while Type 2 represented 40.0% and Type 3 only 11% (Figure 5a). We then grouped participants by their dominant VLY type and examined *Gardnerella* relative abundance and symptom frequency. If a similar number of reads were observed for two or more toxin types in a sample, that sample was classified as being dominated by both types. CLARK-S [30] was used to classify taxa from metagenomic reads and to determine the relative abundance of the taxa comprising the vaginal community profile in each subject. In contrast to what we observed for *vly* read distribution, participants in the Type 1 VLY group exhibited a median relative *Gardnerella* abundance (relative to the whole microbiome of the individual subject) of only 24.1%, while those in the Type 2 VLY group exhibited a significantly higher *Gardnerella* abundance of 62.0% (*p* = 0.0077) (Figure 5b). Subjects in the Type 3 VLY group exhibited the lowest *Gardnerella* median relative abundance at 12.9% (*p* = 0.03). Thus, *Gardnerella* that encode VLY Type 2 tend to exhibit higher proportional abundance than *Gardnerella* strains encoding other VLY types.

### 2.6. Vaginal Symptoms May Be Associated with VLY Type 

**Figure 6:**
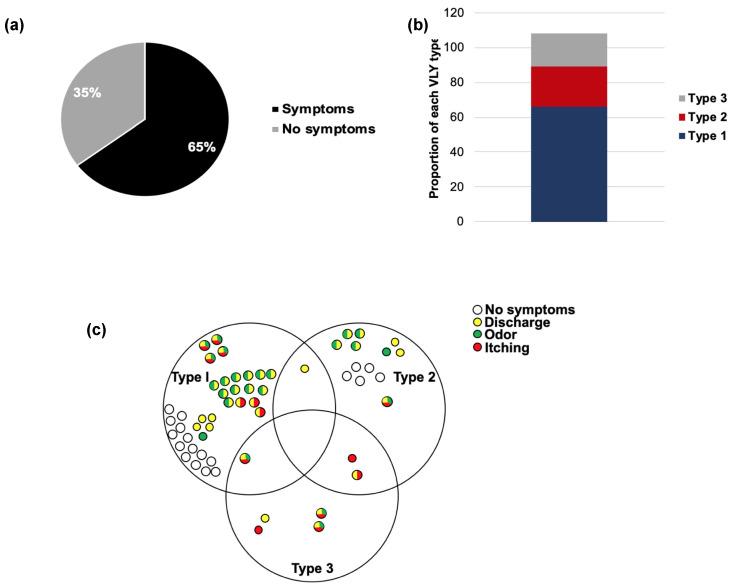
Subjects with type 1 VLY more frequently experience vaginal symptoms. (**a**) Percentage of subjects reporting vaginal symptoms. Subjects reporting “No” for each of itching, odor, or discharge were counted as “No symptoms”. Subjects reporting “Not sure” (*n* = 2) were not counted. “Symptoms” represents the percentage of subjects reporting 1, 2, or 3 symptoms. (**b**) Proportion of each VLY type among symptomatic women. Values do not total to 100% because some samples have >1 dominant VLY type. (**c**) Symptom distribution by VLY type.

Overall, 65% of the subjects reported at least one vaginal symptom (itching, odor, or discharge), while 35% were asymptomatic (Figure 6a). After grouping participants by their VLY type, we observed that a greater number of subjects overall were dominated by Type 1 VLY (*n* = 39) compared to Types 2 (*n* = 16) or 3 (*n* = 7) (Figure 6b). Interestingly, none of the Type 3 VLY-dominant subjects were asymptomatic (Figure 6b,c). Subjects with Type 1 or 3 dominant profiles more frequently reported more than one symptom (48.7% and 57.1%, respectively) compared to subjects with a Type 2 dominant profile (37.5%) (Figure 6c). Almost all reports of itching (92.9%) were by subjects with either Type 1- or 3- dominant VLY (Figure 6c). Thus, Type 1 VLY may be more frequently associated with itching, and Type 3 may be more associated with symptoms in general.

## 3. Discussion

In this study, we compared VLY amino acid sequences across 91 publicly available *Gardnerella* genome sequences. Through comparisons focusing on the conserved undecapeptide and CD59 regions, we distinguished five distinct VLY types that were differentially distributed among the 13 recognized *Gardnerella* species. While Types 1A-C were found in the species groups previously defined [2] as 1–3, 7–10, and 13, VLY Type 2 was only found in species groups 5 and 6. These distributions are consistent with a 2012 study that performed a comparative analysis of 17 *Gardnerella* isolates [28]. The analysis of VLY amino acid sequences of these 17 isolates provided evidence that VLY from clades 1 (species groups 1 and 2), 2 (species group 3), and 3 (species groups 8–10) engaged in recombination between clades, while clade 4 (species groups 5 and 6) retained a unique identity. Our study builds upon this by introducing VLY Type 3 as an additional candidate VLY with a unique identity. It was only identified in a single strain representing species group 12, which does not fall into one of the four previously described clades. Inspection of the undecapeptide and proximal CD59 regions indicate characteristics of both Type 1 and Type 2 VLYs. While only ~1% of the publicly available strains we analyzed encoded the Type 3 toxin, 15% of subjects from the VaHMP cohort had at least 25% of *Gardnerella* reads coming from this species group. Interestingly, all of these samples exhibited less than 20% relative *Gardnerella* abundance (relative to the whole microbiome). Thus, VLY Type 3 warrants more investigation and should be isolated and functionally compared to the other two predominant VLY types.

Alignment of *Gardnerella* strains based on *cpn60* sequences and subsequent mapping of species group and VLY type revealed that ~15% of analyzed strains were devoid of *vly*. While the existence of *Gardnerella* strains lacking the vaginolysin gene has been noted previously, we noticed that species group 4 (*G. piotii*) was particularly enriched in *vly*-deficient strains and that species group 11, represented by a single strain, was also *vly*-deficient [7,30,31]. When we looked at samples containing a large percentage (at least 25%) of either of these two species groups among the total *Gardnerella* population, we observed a wide range (9.5–65%) of relative *Gardnerella* abundance within the total bacterial population of each sample. We also noticed that out of all 62 samples, only one subject was colonized by a single *Gardnerella* species group. All other samples contained more than one species, suggesting that different species may play synergistic roles during colonization. Therefore, although several *vly* null strains of *Gardnerella* exist and may be relatively abundant during BV, this does not dispute an important role for the toxin, as VLY produced by other strains within the community could compensate. In fact, a recent publication by Bohr and colleagues provides evidence of purifying selection in VLY, suggesting that VLY is indeed important for the fitness of *Gardnerella* spp. [32]. Furthermore, in light of the finding that multiple *Gardnerella* species typically coexist in a single host, there may exist a broader interdependence between species so that infection with multiple, distinct species confers an advantage to the bacteria. Future research should be directed at defining the contributions that disparate *Gardnerella* species make to promote community success within the vaginal mucosa. 

To assess whether amino acid differences in VLY types could alter toxin function, we performed in vitro assays using recombinantly produced toxins of the two most common divergent VLY types, Type 1A and Type 2. Similar to a 2012 study that compared the hemolytic activity of five *Gardnerella* strains with divergent VLY sequences [6] in which VLY sequence did not affect the hemolytic activity of VLY, we found that both Type 1A and Type 2 VLY were highly cytotoxic. However, when the vaginal epithelial cell monolayers were pre-incubated with a CD59-specific antibody to block the CD59 receptors, they were significantly more sensitive to the Type 1A VLY. These results could suggest a difference in the CD59 affinity of Type 1A VLY relative to Type 2 VLY or a difference in the dependence of the two VLY types upon the co-receptor. We recently reported that in a stratified, polarized model of the human vaginal epithelium, VLY Type 2 cytotoxicity was limited to the basolateral side. Apical (lumenal) cells were resistant to the toxin [13]. These apical cells also exhibited reduced surface expression of CD59 relative to basolateral cells. Thus, we hypothesized that the observed differences in cytotoxicity between Type 1A and Type 2 toxins could potentiate important differences in the interaction with the host epithelium in vivo. Specifically, Type 1A, if it indeed has an increased affinity for CD59, could be more damaging to the lumenal (apical) side of the vaginal mucosa, which *Gardnerella* spp. directly interacts with in vivo. Because of the reduced CD59 expression on this side, Type 2 VLY, if it has lower affinity for CD59, would elicit less damage. However, a crystallographic analysis of the interaction between VLY and CD59 identified different residues that participated in the bond [11], and these residues were completely conserved between all VLY types (see Supplementary Figure S1). To demonstrate that specific amino acid differences are causal in these observed differences, analysis of single amino acid substitutions within versions of the toxin will be required. Furthermore, as VLY is known to interact with cholesterol and other glycans [8,33], investigation of these molecules should be included in future studies of VLY toxin variants. 

The cytokines altered in response to either toxin were, for the most part, similar. There were some significant differences in cytokine quantities between differentially challenged cells, which could be due to differences in binding, sublethal pore formation, or cell death induced by either toxin. Type 2 VLY induced a stronger IL-8 response than Type 1. If Type 1 VLY is superior at suppressing IL-8-mediated neutrophil chemotaxis, then this could further increase the damage mediated by Type 1-producing species. This could explain our finding that subjects colonized by predominantly Type 1-producing *Gardnerella* more frequently reported symptoms even though subjects colonized by Type 2-producing *Gardnerella* exhibited increased *Gardnerella* abundance. Type 2 VLY could promote bacterial persistence by minimizing damage and assuming a more commensal role. Our findings are in agreement with a recent publication investigating the co-occurrence patterns of the newly classified *Gardnerella* spp. and their associations with vaginal symptoms [29]. Hill et al. observed a strong relationship between higher relative abundance of *G. vaginalis* (Type 1 VLY) and odor and discharge symptoms. This relationship was also observed for *G. swidsinskii* (Type 2 VLY); however, the authors note that this may be due to the significant co-occurrence observed between *G. vaginalis* and *G. swidsinskii*. A similar relationship was not detected for *G. leopoldii* (Type 2 VLY).

In sum, this study highlights the relatively large number of *Gardnerella* species that often co-occur in vaginal microbiomes and reveals the relationship between species and VLY type. Future studies with versions of recombinant VLY that vary in a single amino acid and use of a model system of vaginal epithelial cells that vary in CD59 expression would greatly enhance our understanding of how the toxin functions and interacts with the host.

## 4. Materials and Methods

### 4.1. Clade, Species, and VLY Type Assignment to Available Gardnerella Genomes

In order to extract the VLY and cpn60 sequences from all the *Gardnerella* genomes available on NCBI, we created a profile Hidden Markov Model using HMMER3 for the pfam PF01289 (Thiol_cytolysin protein) and pfam PF00118 (Cpn60_TCP1). All proteins and coding sequences from the 110 complete and draft *Gardnerella* genomes available from NCBI were downloaded. Proteins were searched using HMMsearch to find hits to the PF0289 and PF00118 HMM profiles created above, and the corresponding coding sequences were extracted from each of the genomes. The *Gardnerella* strains from which VLY sequences were extracted (91 strains after removing duplicates) were automatically aligned using MUSCLE [34] and the final alignment was manually inspected. Following a similar process, 91 cpn60 sequences were extracted. The Neighbor-Joining tree using Kimura-2-Parameter algorithm was inferred by using the Phylogeny.fr tool [35,36]. Clade and species designations were assigned manually using schema outlined in Ahmed et al. 2012 and Vaneechoutte et al., 2019, respectively [2,37]. VLY type was assigned based on the scheme described below.

### 4.2. VLY Alignment

Full-length VLY sequences were aligned using Clustal Omega and colored to highlight conserved and divergent sequences [37].

### 4.3. Distinction of VLY Types 

Based on the similarities and differences observed in the sequence alignment, VLY types were first classified based on sequence differences in the CD59 region [10,26]. Type 1 VLY contained aspartate and serine in positions 4 and 6 of the CD59 region, while Type 2 VLY contained asparagine and threonine and Type 3 VLY contained asparagine and serine. The toxin types were then sub-grouped based on sequence differences in the undecapeptide region. Since Types 2 and 3 were uniform in this region, there were no subgroups for these types. Type 1, on the other hand, had either double valine (1A), valine and alanine (1B), or glutamine and valine(1C) at positions −1 and 6 of the undecapeptide region. 

### 4.4. Expression and Purification of Recombinant VLY

Nucleotide sequences for *vly* were extracted from whole-genome sequences (ATCC 14,018 GenBank assembly accession: GCA_003397685.1; AMD: GCA_000176475.1) using Hidden Markov Model. The *vly* gene lacking the signal sequence was amplified from *G. leopolidii* strain AMD (Type 2) and cloned into pET32XT as previously described [38]. *G. vaginalis* strain 14,018 (Type 1A) was similarly amplified and cloned with the following primers: 14018_VLY_FWD (5′-GGAAGGGATCCGCTCCTTCCGCTAAGGATTCTG-3′), 14018_VLY_REV (5′- GGAAGCTCGAGTCAGTCGTTCTTTACAGTTTCAGCAAC-3′). Protein was expressed in *E. coli* strain BL21(DE3) CodonPlus pRIPL (Agilent technologies). One liter cultures were grown to exponential phase (OD_600_ = 0.5) and induced with 1 mM IPTG for 2 h, and the bacteria were collected by centrifugation. Bacteria were lysed in a French pressure cell in 25 mL B-PER™ (Thermo Fisher Scientific, Waltham, MA, USA) containing 1 protease inhibitor tablet (EDTA-free cOmplete™, MilliporeSigma, Burlington, MA USA), and the lysate was cleared by centrifugation and filtration. The protein (VLY) was purified by cobalt affinity chromatography (His-Pur™, Thermo Fisher Scientific) according to manufacturer instructions, eluted in 0.25 M imidazole, and dialyzed against 1X phosphate-buffered saline (PBS) at 4 °C overnight. VLY in 1X PBS was then concentrated using a 30 K centrifugal filter. The affinity tag was left attached. Purified, concentrated protein was then sterile-filtered, and aliquots frozen at −80 °C until needed. The empty vector control was similarly prepared.

### 4.5. Comparison of VLY Monolayer Cytotoxicity

VK2/E6E7 cells [39] were obtained from ATCC and cultured in complete keratinocyte medium (serum-free, 0.1 ng/mL human recombinant epidermal growth factor (EGF), 0.05 mg/mL bovine pituitary extract, 0.4 mM CaCl_2_,). Preparations of either Type 1A rVLY (from strain 14018) or Type 2 rVLY (from strain AMD) containing similar concentrations of the toxin, as determined by visualization of ~73 kDa band (57 kDa VLY plus 6xHis tag) on a NuPAGE 4 to 12% Bis-Tris gel under denaturing conditions, were compared. Protein preparations were diluted to 1 µg/mL in VK2 culture medium without antibiotics, and endotoxin levels were assayed using the ToxinSensor^TM^ gel clot endotoxin assay kit. This kit detects endotoxin levels as low as 0.25 EU. The rVLY and empty vector preparations used in these assays contained undetectable levels of endotoxin. One hundred microliters of the diluted rVLY, empty vector control, or medium alone was added to approximately 3 × 10^4^ adherent VK2 cells in each of 3 individual wells of 96-well plates. After 4 h of co-incubation at 37 °C, the media were removed and 100 μl of a 1:1 solution of 0.4% trypan blue and PBS was added to each well and incubated at 37 °C for 1 min. The trypan blue was removed, and the monolayers were imaged by light microscopy. To assess the percentage of cells staining with trypan blue, 1000 cells were counted for each sample. Some monolayers were pretreated with 1:1000 anti-CD59 (Novus Biologicals, St. Charles, MO, USA) in cell culture medium for 1 h. Percentage trypan blue staining was calculated by diving the number of blue-stained cells in an image by the total number of cells and multiplying by 100. The means were compared by using a Student’s t-test.

### 4.6. VK2 Monolayer Cytokine Analysis

Five hundred microliters of 1 µg/mL either Type 1A or Type 2 rVLY in complete keratinocyte medium were added to approximately 4 × 10^5^ adherent VK2 cells per well of 24-well plates in triplicate and then incubated aerobically in 5% CO_2_ at 37 °C. After 19 h of co-incubation, media were collected from the wells, and any cells were removed by centrifugation. Spent medium was analyzed for relative cytokine concentrations using a Bio-Plex Pro Human 27-Plex Assay. Assays were performed according to manufacturer’s instructions, and plates were analyzed by the Bio-Plex MAGPIX Multiplex Reader (BIO-RAD, Hecules, CA, USA). Cytokine concentrations were quantified by comparing sample values to a standard curve. As a default, the standard curve of each cytokine was fit with a 5-parameter logistic model. For each cytokine, kernel density estimation (with default bandwidth parameter in JMP 15) was used to fit a smooth distribution to the relative concentrations. After the data were smoothed, the 95th percentile of the fitted distribution was used to impute out-of-range above (OOR>) values and the 5th percentile was used for out-of-range below (OOR<) values. If the 5th percentile was less than zero, then zero was used as the imputed value. 

While 27 cytokines were assayed, only those with levels >10 pg/mL under at least one condition are presented in Figure 2. Means were compared using a one-way ANOVA with a post hoc Tukey HSD test.

### 4.7. VLY Type Metagenomic Data Analysis

We analyzed 64 samples from the Vaginal Human Microbiome Project (VaHMP) cohort [26]. In this study, we collected more than 40,000 vaginal, cervical, introital and buccal swab samples cross-sectionally from a racially diverse cohort of 3815 female subjects visiting VCU Medical Center. Samples were collected with BD BBL CultureSwab EZ swabs. Swabs were processed immediately using MoBio Powersoil kit as described by the manufacturer. DNA samples were stored at −80 °C. DNA libraries were prepared using Illumina Truseq DNA library prep kit and sequenced on our Illumina HiSeq 2000 (2 × 100b PE). Samples were sequenced individually in each lane, and 5 × 10^8^ paired-end reads were obtained per sample. For whole shotgun metagenomic data pre-processing, raw sequence data were demultiplexed into sample-specific fastq files using *bcl2fastq* conversion software from Illumina. The pre-processing of raw paired-end sequence data was performed using Trimmomatic, by removing Illumina specific adapters from the reads and trimming the 5′ end for quality using a sliding-window approach (average Q20 over a window of 4 b). Reads shorter than 70 bp after trimming were discarded. This was followed by removal of contaminant reads of vector and human origin by aligning against the UniVec database (https://www.ncbi.nlm.nih.gov/tools/vecscreen/univec/) and the hg19 build of the human genome using bwa. Two samples had fewer than 100,000 high-quality reads and were removed from further downstream analysis. Taxonomic classification of the metagenomics sequence data was performed on high-quality non-human reads by alignment to a custom database of bacterial genomes using CLARK-S, a discriminative space k-mer-based approach. Bacterial genomes were downloaded from the NCBI Reference Sequence Database, accessed in March 2017, supplemented with our in-house genome assembly of Lachnospiraceae_BVAB1. Species abundances were estimated by normalizing the assigned read counts to species genome size. To determine whether variants in addition to those in the NCBI sequence data occurred in the metagenomic dataset, high-quality reads were assembled with SPAdes ver 3.8.0 [40] using the “-meta” option. Contig sequences were annotated using Prokka [41].

### 4.8. Gardnerella Spp. Cpn60 Database

In order to create a comprehensive *cpn60* database, we first created a profile Hidden Markov Model using HMMER3 for the pfam PF00118 protein family (TCP-1/cpn60 chaperonin family). This method uses a homology detection approach by comparing sequence reads to HMM profile. All proteins and coding sequences from complete and draft from 105 *Gardnerella* genomes available from NCBI were downloaded. Proteins were searched using HMMsearch to find hits to the PF00118 model created above, and the corresponding coding sequences were extracted from each of the genomes. This *cpn60* database was used to taxonomically classify the *Gardnerella* reads from the metagenomic data to the species/strain levels.

### 4.9. VLY Type Distribution among Study Participants

The number of *vly* reads of each type in a given sample was used to determine the “dominant” VLY type of the sample. The VLY type exhibiting a clear majority of the total *vly* reads for a sample (representing ≥50% of all *vly* reads) was designated as the dominant type. If two VLY types were equally represented and together made up a majority of the total *vly* reads in a sample, this sample was said to be dominated by both types and placed into both VLY type groups. The number of reads of a given VLY type across all samples was divided by the total number of *vly* reads across all samples and multiplied by 100 to give a percent value. Of the 62 samples classified as having at least 100,000 high-quality reads, two lacked any *vly* reads and were removed from further downstream analysis. 

### 4.10. Relative Bacterial Abundance in Subjects with Different VLY Types

16S rRNA sequencing reads were used to determine the relative abundance of different bacterial species in each sample. After sample grouping by VLY type, the median relative abundance of each bacterial species was calculated. As relative abundance data were not normally distributed, a non-parametric Wilcoxon test was used to test for statistically significant differences in the median relative abundance values between VLY type groups. 

### 4.11. Statistical Analysis

All data were assessed for normality by inspection of a Normal Quantile Plot. Equality of variance was assessed using the Brown-Forsythe test. The tests used for the comparison of mean or median values are described in each relevant section. All statistical analyses were performed using JMP Pro 14.0 software (SAS Institute Inc., Cary, NC, USA). *p*-values of less than 0.05 were considered significant. Fold changes of 2 or higher were considered biologically relevant.

## Data Availability

Data from the VaHMP has been deposited under dbGAP Study Accession phs000256.v3.p2. Raw metatranscriptomic sequences from the MOMS-PI project are available at NCBI’s controlled-access dbGaP (Study Accession: phs001523.v1.p1).

## Supplementary Materials

The following are available online at https://www.mdpi.com/2076-0817/10/2/86/s1, Figure S1: Alignment of VLY amino acid sequences. Figure S2: Alignment of VLY undecapeptide and CD59 regions reveals distinct VLY types. Figure S3: Equivalent purity and concentration of Type 1A and Type 2 rVLY. Figure S4: Relative abundance of bacterial species, *Gardnerella* genomic species, vly types, and inerolysin in VHMP samples.
