# Supplementary material for: Sequence Comparison of Vaginolysin from Different Gardnerella Species
Source: Pathogens. 2021 Jan 20;10(2):86. doi: 10.3390/pathogens10020086 (PMC7909246; doi:10.3390/pathogens10020086)
Supplement: Supplementary file 1 [file pathogens-10-00086-s001.pdf]

Figure S1. Alignment of VLY amnio acid sequences

```
                                  1        10        20        30        40        50        60        70
WP_009567818.1_Sintermedius       MKTKQNIARKLS.RVVLLSTLVLSSAAPISAAFAETPTKPKAAQTEK...KPE..KKPENSNSEAAKKALNDYIWGL
WP_006730404.1_Liners             MNKKGLRK....IIYASSLALLELSLVTATKPAQAATNTEPKTAIVAKHNTEEPMSVNDYLLGLDYDSQAVLTRQGD
GCA_003397685_ATCC14018           MKSTKFYR.NAAMLLLAGATIVPQCLAAPAMAAPSAKDSEPATSCAA...KKD..SLNNYLWDLQYDKTNILARHGE
GCA_000159155_ATCC14019           MKSTKFYR.NAAMLLLAGATIVPQCLAAPAMAAPSAKDSEPATSCAA...KKD..SLNNYLWDLQYDKTNILARHGE
GCA_900637625_NCTC10287           MKSTKFYR.NAAMLLLAGATIVPQCLAAPAMAAPSAKDSEPATSCAA...KKD..SLNNYLWDLQYDKTNILARHGE
GCA_001042655_JCM11026            MKSTKFYR.NAAMLLLAGATIVPQCLAAPAMAAPSAKDSEPATSCAA...KKD..SLNNYLWDLQYDKTNILARHGE
GCA_900105405_DSM4944             MKSTKFYR.NAAMLLLAGATIVPQCLAAPAMAAPSAKDSEPATSCAA...KKD..SLNNYLWDLQYDKTNILARHGE
GCA_003034925_ATCC49145           MKSTKFYR.NAAMLLLAGATIVPQCLAAPAMAAPSAKDSEPATSCAA...KKD..SLNNYLWDLQYDKTNILARHGE
GCA_003812765_FDAARGOS_568        MKSTKFYR.NAAMLLLAGATIVPQCLAAPAMAAPSAKDSEPATSCAA...KKD..SLNNYLWDLQYDKTNILARHGE
GCA_002206225_FDAARGOS_296        MKSTKFYR.NAAMLLLAGATIVPQCLAAPAMAAPSAKDSEPATSCAA...KKD..SLNNYLWDLQYDKTNILARHGE
GCA_002861975_UMB0298             MKSTKFYR.NAAMLLLAGATIVPQCLAAPAMAAPSAKDSEPATSCAA...KKD..SLNNYLWDLQYDKTNILARHGE
GCA_002861965_UMB0386             MKSTKFYR.NAAMLLLAGATIVPQCLAAPAMAAPSAKDSEPATSCAA...KKD..SLNNYLWDLQYDKTNILARHGE
GCA_003397605_UGent25.49          MNNTKFYR.NAAMLLLAGATIIPQCLAAPAMAAPAAKDSEPTASCAA...KKD..SLNNYLWDLQYDKTNILARHGE
GCA_003408745_GH015               MKSTKFYR.NAAMLLLAGATIVPQCLAAPAMAAPSAKDSEPATSCAA...KKD..SLNNYLWDLQYDKTNILARHGE
GCA_002861945_UMB0770             MKSTKFYR.NAAMLLLAGATIVPQCLAAPAMAAPSAKDSEPATSCAA...KKD..SLNNYLWDLQYDKTNILARHGE
GCA_001049785_3549624             MKSTKFYR.NAAMLLLAGATIVPQCLAAPAMAAPSAKDSEPATSCAA...KKD..SLNNYLWDLQYDKTNILARHGE
GCA_000213955_HMP9231             MKSTKFYR.NAAMLLLAGATIVPQCLAAPAMAAPSAKDSEPATSCAA...KKD..SLNNYLWDLQYDKTNILARHGE
GCA_002861925_UMB0775             MKSTKFYR.NAAMLLLAGATIVPQCLAAPAMAAPSAKDSEPATSCAA...KKD..SLNNYLWDLQYDKTNILARHGE
GCA_003426545_NR001               MKSTKFYR.NAAMLLLAGATIIPQCLAAPAMAAPAAKDSEPTASCAA...KKD..SLNNYLWDLQYDKTNILARHGE
GCA_003585655_NR038               MKSTKFYR.NAAMLLLAGATIIPQCLAAPAMAAPAAKDSEPTASCAA...KKD..SLNNYLWDLQYDKTNILARHGE
GCA_002894105_DNF01149            MKSTKFYR.NAAMLLLAGATIVPQCLAAPAMAAPSAKDSEPATSCAA...KKD..SLNNYLWDLQYDKTNILARHGE
GCA_000263435_284V                MKSTKFYR.NAAMLLLAGATIVPQCLAAPAMAAPSAKDSEPATSCAA...KKD..SLNNYLWDLQYDKTNILARHGE
GCA_003426285_WP023               MKSTKFYR.NAAMLLLAGATIVPQCLAAPAMAAPSAKDSEPATSCAA...KKD..SLNNYLWDLQYDKTNILARHGE
GCA_002884835_UMB0768             MKSTKFYR.NAAMLLLAGATIVPQCLAAPAMAAPSAKDSEPATSCAA...KKD..SLNNYLWDLQYDKTNILARHGE
GCA_003397665_UGent09.07          MKSTKFYR.NAAMLLLAGATIVPQCLAAPAMAAPSAKDSEPATSCAA...KKD..SLNNYLWDLQYDKTNILARHGE
GCA_000263555_0288E               MKSTKFYR.NAAMLLLAGATIVPQCLAAPAMAAPSAKDSEPATSCAA...KKD..SLNNYLWDLQYDKTNILARHGE
GCA_000414685_JCP7276             MKSTKFYR.NAAMLLLAGATIVPQCLAAPAMAAPSAKDSEPATSCAA...KKD..SLNNYLWDLQYDKTNILARHGE
GCA_000414645_JCP7672             MNNTKFYR.NAAMLLLAGATIIPQCLAAPAMAAPAAKDSEPTASCAA...KKD..SLNNYLWDLQYDKTNILARHGE
GCA_003397755_UGent09.01          MNNTKFYR.NAAMLLLAGATIIPQCLAAPAMAAPAAKDSEPTASCAA...KKD..SLNNYLWDLQYDKTNILARHGE
GCA_000263515_00703C2mash         MKSSKFYR.NAAMLLLAGATIVPQCLAAPAMAAPSAKDSEPATSCAA...KKD..SLNNYLWDLQYDKTNILARHGE
GCA_000263615_00703Bmash          MNNTKFYR.NAAMLLLAGATIIPQCLAAPAMAAPAAKDSEPTASCAA...KKD..SLNNYLWDLQYDKTNILARHGE
GCA_003408835_N144                MKSSKFYR.NAAMLLLAGATIVPQCLAAPAMAAPSAKDSEPATSCAA...KKD..SLNNYLWDLQYDKTNILARHGE
GCA_001546445_GED7275B            MKSTKFYR.NAAMLLLAGATIIPQCLAVPAMATPASKDPEPAPSCAA...NKN..ALNDYLWDLQYDKTSILARHGE
GCA_000414605_JCP8017A            MNNTKFYR.NAAMLLLAGATIIPQCLAAPAMAAPAAKDSEPTASCAA...KKD..SLNNYLWDLQYDKTNILARHGE
GCA_000414585_JCP8017B            MNNTKFYR.NAAMLLLAGATIIPQCLAAPAMAAPAAKDSEPTASCAA...KKD..SLNNYLWDLQYDKTNILARHGE
GCA_000414665_JCP7659             MKSSKFYR.NAAMLLLAGATIVPQCLAAPAMAAPSAKDSEPATSCAA...KKD..SLNNYLWDLQYDKTNILARHGE
GCA_003408775_N160                MKNSKFYR.NAAMLLLAGATIIPQCLAVPAMAAPSAKDSEPATSCAA...KKD..SLNNYLWDLQYDKTNILARHGE
GCA_000263655_6119V5              MKNSKFYR.NAAMLLLAGATIIPQCLAVPAMAAPSAKDSEPATSCAA...KKD..SLNNYLWDLQYDKTNILARHGE
GCA_000263535_75712               MKSTKFYR.NAAMLLLAGATIVPQCLAAPAMAAPSAKDSEPATSCAA...KKD..SLNNYLWDLQYDKTNILARHGE
GCA_003585755_NR039               MKSTKFYR.NAAMLLLAGATIVPQCLAAPAMAAPSAKDSEPATSCAA...KKD..SLNNYLWDLQYDKTNILARHGE
GCA_002862045_UMB0233             MNNTKFYR.NAAMLLLAGATIIPQCLAAPAMAAPAAKDSEPTASCAA...KKD..SLNNYLWDLQYDKTNILARHGE
GCA_000214315_315-A               MNNTKFYR.NAAMLLLAGATIIPQCLAAPAMAAPAAKDSEPTASCAA...KKD..SLNNYLWDLQYDKTNILARHGE
GCA_002862005_UMB0032B            MNNTKFYR.NAAMLLLAGATIIPQCLAAPAMAAPAAKDSEPTASCAA...KKD..SLNNYLWDLQYDKTNILARHGE
GCA_002862015_UMB0032A            MNNTKFYR.NAAMLLLAGATIIPQCLAAPAMAAPAAKDSEPTASCAA...KKD..SLNNYLWDLQYDKTNILARHGE
GCA_002861165_UMB0061             MKSTKFYR.NAAMLLLAGATIVPQCLAAPAMAAPSAKDSEPATSCAA...KKD..SLNNYLWDLQYDKTNILARHGE
GCA_001660755_18-4                MKSTKFYR.NAAMLLLAGATIVPQCLAAPAMAAPSAKDSEPATSCAA...KKD..SLNNYLWDLQYDKTNILARHGE
GCA_000165635_41V                 MNNTKFYR.NAAMLLLAGATIIPQCLAAPAMAAPAAKDSEPTASCAA...KKD..SLNNYLWDLQYDKTNILARHGE
GCA_000263475_55152               MNNTKFYR.NAAMLLLAGATIIPQCLAAPAMAAPAAKDSEPTASCAA...KKD..SLNNYLWDLQYDKTNILARHGE
GCA_000263495_1400E               MNNTKFYR.NAAMLLLAGATIIPQCLAAPAMAAPAAKDSEPTASCAA...KKD..SLNNYLWDLQYDKTNILARHGE
GCA_000414525_JCP8108             MNNTKFYR.NAAMLLLAGATIIPQCLAAPAMAAPAAKDSEPTASCAA...KKD..SLNNYLWDLQYDKTNILARHGE
GCA_002861905_UMB0830             MNNTKFYR.NAAMLLLAGATIIPQCLAAPAMAAPAAKDSEPTASCAA...KKD..SLNNYLWDLQYDKTNILARHGE
GCA_003369895_N101                MNNTKFYR.NAAMLLLAGATIIPQCLAAPAMAAPAAKDSEPTASCAA...KKD..SLNNYLWDLQYDKTNILARHGE
GCA_003369935_N153                MNNTKFYR.NAAMLLLAGATIIPQCLAAPAMAAPAAKDSEPTASCAA...KKD..SLNNYLWDLQYDKTNILARHGE
GCA_003369965_N95                 MNNTKFYR.NAAMLLLAGATIIPQCLAAPAMAAPAAKDSEPTASCAA...KKD..SLNNYLWDLQYDKTNILARHGE
GCA_000414625_JCP7719             MNNTKFYR.NAAMLLLAGATIIPQCLAAPAMAAPAAKDSEPTASCAA...KKD..SLNNYLWDLQYDKTNILARHGE
GCA_000414485_JCP8151B            MNNTKFYR.NAAMLLLAGATIIPQCLAAPAMAAPAAKDSEPTASCAA...KKD..SLNNYLWDLQYDKTNILARHGE
GCA_000263635_00703Dmash          MKNSKFYR.NAAMLLLAGATIIPQCLAAPAMAAPSAKDSEPATSCAA...KKD..SLNNYLWDLQYDKTNILARHGE
GCA_000165615_101                 MKNSKFYR.NAAMLLLAGATIIPQCLASPAMAAPSAKDSEPATSCAA...KKD..SLNNYLWDLQYDKTNILARHGE
GCA_002884775_UMB1686             MKNTKFYR.NAAMLLLAGATIIPQCLAAPAMAAPSAKDSEPATSCAA...KKD..SLNNYLWDLQYDKTNILARHGE
GCA_000263595_1500E               MKNTKFYR.NAAMLLLAGATIIPQCLAAPAMAAPSAKDSEPATSCAA...KKD..SLNNYLWDLQYDKTNILARHGE
GCA_000414445_JCP8481B            MKKIKFYR.NAAMLLLAGATILPQGLAAPAMAAPAPKAPEPTPSCAA...SKD..ALNNYLWDLQYDKTNILARHGE
GCA_000414465_JCP8481A            MKKIKFYR.NAAMLLLAGATILPQGLAAPAMAAPAPKAPEPTPSCAA...SKD..ALNNYLWDLQYDKTNILARHGE
GCA_001660735_23-12               MNNTKFYR.NAAMLLLAGATIIPQCLAAPAMAAPAAKDSEPTASCAA...KKD..SLNNYLWDLQYDKTNILARHGE
GCA_003408845_NR010               MKNTKFYR.NAAMLLLAGATIIPQCLAAPAMAAPAAKDSEPTTSCAV...KKD..SLNNYLWDLQYDKTNILARHGE
GCA_003426445_NR037               MKSTKFYR.NAAMLLLAGATIVPQCLAAPAMAAPSAKDSEPATSCAA...KKD..SLNNYLWDLQXXXXXXXXXXXX
GCA_002896555_KA00225             MVNSKFYR.NVAMMLLAGATVIPQGLAIPAMASPAEKTPENN.SCAV...KKS..ALNDYLWDLKYDKTNILARHGE
GCA_001546485_PSS_7772B           MKKIKFYR.NAAMLLLAGATILPQGLAAPAMAAPAPKAPEPTPSCAA...SKD..ALNNYLWDLQYDKTNILARHGE
GCA_000025205_409-05              MKSTKFYR.NAAMLLLAGATIIPQCLAVPAMADSSAKPSAPAASCAA...SKD..SLNNYLWDLHYDKTNILARHGE
GCA_000176495_5-1                 MKSTKFYR.NAAMLLLAGATIIPQCLAVPAMADSSAKPSAPAASCAA...SKD..SLNNYLWDLHYDKTNILARHGE
GCA_001953155_GV37                MKSTKFYR.NAAMLLLAGATIIPQCLAVPAMADSSAKPSAPAASCAA...SKD..SLNNYLWDLHYDKTNILARHGE
GCA_002884795_UMB1642             MKSTKFYR.NAAMLLLAGATIIPQCLAVPAMADSSAKPSAPAASCAA...SKD..SLNNYLWDLHYDKTNILARHGE
GCA_002884855_UMB0170             MKSTKFYR.NAAMLLLAGATIIPQCLAVPAMADSSAKPSAPAASCAA...SKD..SLNNYLWDLHYDKTNILARHGE
GCA_002884875_UMB0264             MKSTKFYR.NAAMLLLAGATIIPQCLAVPAMADSSAKPSAPAASCAA...SKD..SLNNYLWDLHYDKTNILARHGE
GCA_003397705_GS9838-1            MKSTKFYR.NAAMLLLAGATIIPQCLAVPAMADSSAKPSAPAASCAA...SKD..SLNNYLWDLHYDKTNILARHGE
GCA_003397745_GS10234             MKSTKFYR.NAAMLLLAGATIIPQCLAVPAMADSSAKPSAPAASCAA...SKD..SLNNYLWDLHYDKTNILARHGE
GCA_003408815_N72                 MKSTKFYR.NAAMLLLAGATIIPQCLAVPAMADSSAKPSAPAASCAA...SKD..SLNNYLWDLHYDKTNILARHGE
GCA_002861125_UMB0912             MKSTKFYR.NAAMLLLAGATIIPQCLAVPAMADSSTKPSAPAASCAA...SKD..SLNNYLWDLHYDKTNILARHGE
GCA_002861145_UMB0913             MKSTKFYR.NAAMLLLAGATIIPQCLAVPAMADSSTKPSAPAASCAA...SKD..SLNNYLWDLHYDKTNILARHGE
GCA_003397635_UGent09.48          MKSTKFYR.NAAMLLLAGATIIPQCLAVPAMADSSTKPSAPAASCAA...SKD..SLNNYLWDLHYDKTNILARHGE
GCA_000263575_6420B               MKSTKFYR.NAAMLLLAGATIIPQCLAVPAMADSSTKPSAPAASCAA...SKD..SLNNYLWDLHYDKTNILARHGE
GCA_000176475_AMD                 MKSTKFYR.NAAMLLLAGATIIPQCLAVPAMADSSTKPSAPAASCAA...SKD..SLNNYLWDLHYDKTNILARHGE
GCA_003293675_UGent06.41          MKSTKFYR.NAAMLLLAGATIIPQCLAVPAMADSSTKPSAPAASCAA...SKD..SLNNYLWDLHYDKTNILARHGE
GCA_002862065_UMB0682             MKSTKFYR.NAAMLLLAGATIIPQCLAVPAMADSSAKPSAPAASCAA...SKD..SLNNYLWDLHYDKTNILARHGE
GCA_001563665_CMW7778B            MVNSKFYR.SVAMMLLAGATIIPQGLALPAMAAPAET.PEND.SCAV...KKD..ALNDYLWDLKYDKTNILARHGE
```

```
                                          80        90        100       110       120       130       140
WP_009567818.1_Sintermedius   QYDKLNILTHQ.GEKLKNHSSREAFHRPGEYVVIEKKKQSISNATSKLSVSSANDDRIFPGALLKADQSLLENLPTL
WP_006730404.1_Liners         MLTNHFAHETTTDNNGRFVVVEKQKKTISNSTSSISVTTANDSRVFAGALFKADNNLIENNPTLVSLRRAPITVSVD
GCA_003397685_ATCC14018       TIENKFSSDSF.NKNGEFVVVEHQKKNITNTTSNLSVTSANDDRVYPGALFRADKNLMDNMPSLISANRAPITLSVD
GCA_000159155_ATCC14019       TIENKFSSDSF.NKNGEFVVVEHQKKNITNTTSNLSVTSANDDRVYPGALFRADKNLMDNMPSLISANRAPITLSVD
GCA_900637625_NCTC10287       TIENKFSSDSF.NKNGEFVVVEHQKKNITNTTSNLSVTSANDDRVYPGALFRADKNLMDNMPSLISANRAPITLSVD
GCA_001042655_JCM11026        TIENKFSSDSF.NKNGEFVVVEHQKKNITNTTSNLSVTSANDDRVYPGALFRADKNLMDNMPSLISANRAPITLSVD
GCA_900105405_DSM4944         TIENKFSSDSF.NKNGEFVVVEHQKKNITNTTSNLSVTSANDDRVYPGALFRADKNLMDNMPSLISANRAPITLSVD
GCA_003034925_ATCC49145       TIENKFSSDSF.NKNGEFVVVEHQKKNITNTTSNLSVTSANDDRVYPGALFRADKNLMDNMPSLISANRAPITLSVD
GCA_003812765_FDAARGOS_568    TIENKFSSDSF.NKNGEFVVVEHQKKNITNTTSNLSVTSANDDRVYPGALFRADKNLMDNMPSLISANRAPITLSVD
GCA_002206225_FDAARGOS_296    TIENKFSSDSF.NKNGEFVVVEHQKKNITNTTSNLSVTSANDDRVYPGALFRADKNLMDNMPSLISANRAPITLSVD
GCA_002861975_UMB0298         TIDNKFSSDSF.NKNGEFVVVEHQKKNITNTTSNLSVTSANDDRVYPGALFRADKNLMDNIPSLISANRAPITLSVD
GCA_002861965_UMB0386         TIDNKFSSDSF.NKNGEFVVVEHQKKNITNTTSNLSVTSANDDRVYPGALFRADKNLMDNIPSLISANRAPITLSVD
GCA_003397605_UGent25.49      TIDNKFSSDSF.NKGDEFVVVEHQKKNITNTTSNLSVTSANDDRVYPGALFRADQNLMDNMPSLISANRAPITLSVD
GCA_003408745_GH015           TIENKFSSDSF.NKNGEFVVVEHQKKNITNTTSNLSVTSANDDRVYPGALFRADKNLMDNMPSLISANRAPITLSVD
GCA_002861945_UMB0770         TIENKFSSDSF.NKNGEFVVVEHQKKNITNTTSNLSVTSANDDRVYPGALFRADKNLMDNMPSLISANRAPITLSVD
GCA_001049785_3549624         TIENKFSSDSF.NKNGEFVVVEHQKKNITNTTSNLSVTSANDDRVYPGALFRADKNLMDNMPSLISANRAPITLSVD
GCA_000213955_HMP9231         TIENKFSSDSF.NKNGEFVVVEHQKKNITNTTSNLSVTSANDDRVYPGALFRADKNLMDNMPSLISANRAPITLSVD
GCA_002861925_UMB0775         TIENKFSSDSF.NKNGEFVVVEHQKKNITNTTSNLSVTSANDDRVYPGALFRADKNLMDNMPSLISANRAPITLSVD
GCA_003426545_NR001           TIDNKFSSDSF.DKGGEFVVVEHQKKNITNTTSNLSVTSANNDRVYPGALFRADQNLMDNMPSLISANRGSITLSVD
GCA_003585655_NR038           TIDNKFSSDSF.DKGGEFVVVEHQKKNITNTTSNLSVTSANNDRVYPGALFRADQNLMDNMPSLISANRGSITLSVD
GCA_002894105_DNF01149        TIENKFSSDSF.NKNGEFVVVEHQKKNITNTTSNLSVTSANDDRVYPGALFRADKNLMDNMPSLISANRAPITLSVD
GCA_000263435_284V            TIENKFSSDSF.NKNGEFVVVEHQKKNITNTTSNLSVTSANDDRVYPGALFRADKNLMDNMPSLISANRAPITLSVD
GCA_003426285_WP023           TIENKFSSDSF.NKNGEFVVVEHQKKNITNTTSNLSVTSANDDRVYPGALFRADKNLMDNMPSLISANRAPITLSVD
GCA_002884835_UMB0768         TIENKFSSDSF.NKNGEFVVVEHQKKNITNTTSNLSVTSANDDRVYPGALFRADKNLMDNMPSLISANRAPITLSVD
GCA_003397665_UGent09.07      TIENKFSSDSF.NKNGEFVVVEHQKKNITNTTSNLSVTSANDDRVYPGALFRADKNLMDNMPSLISANRAPITLSVD
GCA_000263555_0288E           TIENKFSSDSF.NKNGEFVVVEHQKKNITNTTSNLSITSANNDRVYPGALFRADQNLMDNMPSLISANRGSITLSVD
GCA_000414685_JCP7276         TIENKFSSDSF.NKNGEFVVVEHQKKNITNTTSNLSITSANNDRVYPGALFRADQNLMDNMPSLISANRGSITLSVD
GCA_000414645_JCP7672         TIDNKFSSDSF.NKGDEFVVVEHQKKNITNTTSNLSVTSANNDRVYPGALFRADQNLMDNMPSLISANRGSITLSVD
GCA_003397755_UGent09.01      TIDNKFSSDSF.NKGDEFVVVEHQKKNITNTTSNLSVTSANDDRVYPGALFRADQNLMDNMPSLISANRAPITLSVD
GCA_000263515_00703C2mash     TIDNKFSSDSF.NKNGEFVVVEHQKKNITNTTSNLSVTSANDDRVYPGALFRADKNLMDNMPSLISANRAPITLSVD
GCA_000263615_00703Bmash      TIDNKFSSDSF.NKGDEFVVVEHQKKNITNTTSNLSVTSANDDRVYPGALFRADQNLMDNMPSLISANRAPITLSVD
GCA_003408835_N144            TIDNKFSSDSF.NKNGEFVVVEHQKKNITNTTSNLSVTSANDDRVYPGALFRADKNLMDNMPSLISANRAPITLSVD
GCA_001546445_GED7275B        TIDNKFSSDSF.DKGGEFVVVEHQKKNITNTTSNLSVTSANNDRVYPGALFRADQNLMDNMPSLISANRGSITLSID
GCA_000414605_JCP8017A        TIDNKFSSDSF.NKGDEFVVVEHQKKNITNTTSNLSVTSANDDRVYPGALFRADQNLMDNMPSLISANRAPITLSVD
GCA_000414585_JCP8017B        TIDNKFSSDSF.NKGDEFVVVEHQKKNITNTTSNLSVTSANDDRVYPGALFRADQNLMDNMPSLISANRAPITLSVD
GCA_000414665_JCP7659         TIDNKFSSDSF.NKNGEFVVVEHQKKNITNTTSNLSVTSANDDRVYPGALFRADKNLMDNMPSLISANRAPITLSVD
GCA_003408775_N160            TIDNKFSSDSF.NKNGEFVVVEHQKKNITNTTSNLSVTSANDDRVYPGALFRADKNLMDNMPSLISANRAPITLSVD
GCA_000263655_6119V5          TIDNKFSSDSF.NKNGEFVVVEHQKKNITNTTSNLSVTSANDDRVYPGALFRADKNLMDNMPSLISANRAPITLSVD
GCA_000263535_75712           TIENKFSSDSF.NKNGEFVVVEHQKKNITNTTSNLSVTSANDDRVYPGALFRADKNLMDNMPSLISANRAPITLSVD
GCA_003585755_NR039           TIENKFSSDSF.NKNGEFVVVEHQKKNITNTTSNLSVTSANDDRVYPGALFRADKNLMDNMPSLISANRAPITLSVD
GCA_002862045_UMB0233         TIDNKFSSDSF.NKGDEFVVVEHQKKNITNTTSNLSVTSANDDRVYPGALFRADQNLMDNMPSLISGNRAPITLSVD
GCA_000214315_315-A           TIDNKFSSDSF.NKGDEFVVVEHQKKNITNTTSNLSVTSANDDRVYPGALFRADQNLMDNMPSLISANRAPITLSVD
GCA_002862005_UMB0032B        TIDNKFSSDSF.NKGDEFVVVEHQKKNITNTTSNLSVTSANDDRVYPGALFRADQNLMDNMPSLISANRAPITLSVD
GCA_002862015_UMB0032A        TIDNKFSSDSF.NKGDEFVVVEHQKKNITNTTSNLSVTSANDDRVYPGALFRADQNLMDNMPSLISANRAPITLSVD
GCA_002861165_UMB0061         TIENKFSSDSF.NKNGEFVVVEHQKKNITNTTSNLSVTSANDDRVYPGALFRADKNLMDNMPSLISANRAPITLSVD
GCA_001660755_18-4            TIENKFSSDSF.NKNGEFVVVEHQKKNITNTTSNLSVTSANDDRVYPGALFRADKNLMDNMPSLISANRAPITLSVD
GCA_000165635_41V             TIDNKFSSDSF.DKGGEFVVVEHQKKNITNTTSNLSVTSANNDRVYPGALFRADQNLMDNMPSLISANRAPITLSVD
GCA_000263475_55152           TIDNKFSSDSF.DKGGEFVVVEHQKKNITNTTSNLSVTSANNDRVYPGALFRADQNLMDNMPSLISANRAPITLSVD
GCA_000263495_1400E           TIDNKFSSDSF.DKGGEFVVVEHQKKNITNTTSNLSVTSANNDRVYPGALFRADQNLMDNMPSLISANRAPITLSVD
GCA_000414525_JCP8108         TIDNKFSSDSF.DKGGEFVVVEHQKKNITNTTSNLSVTSANNDRVYPGALFRADQNLMDNMPSLISANRAPITLSVD
GCA_002861905_UMB0830         TIDNKFSSDSF.NKGDEFVVVEHQKKNITNTTSNLSVTSANDDRVYPGALFRADQNLMDNMPSLISANRAPITLSVD
GCA_003369895_N101            TIDNKFSSDSF.NKGDEFVVVEHQKKNITNTTSNLSVTSANDDRVYPGALFRADQNLMDNMPSLISANRAPITLSVD
GCA_003369935_N153            TIDNKFSSDSF.NKGDEFVVVEHQKKNITNTTSNLSVTSANDDRVYPGALFRADQNLMDNMPSLISANRAPITLSVD
GCA_003369965_N95             TIDNKFSSDSF.NKGDEFVVVEHQKKNITNTTSNLSVTSANDDRVYPGALFRADQNLMDNMPSLISANRAPITLSVD
GCA_000414625_JCP7719         TIDNKFSSDSF.NKGDEFVVVEHQKKNITNTTSNLSVTSANDDRVYPGALFRADQNLMDNMPSLISANRAPITLSVD
GCA_000414485_JCP8151B        TIDNKFSSDSF.NKGDEFVVVEHQKKNITNTTSNLSVTSANDDRVYPGALFRADQNLMDNMPSLISANRAPITLSVD
GCA_000263635_00703Dmash      TIDNKFSSDSF.NKGDEFVVVEHQKKNITNTTSNLSVTSANDDRVYPGALFRADKNLMDNMPSLISANRAPITLSVD
GCA_000165615_101             TIDNKFSSDSF.NKGDEFVVVEHQKKNITNTTSNLSVTSANDDRVYPGALFRADKNLMDNMPSLISANRAPITLSVD
GCA_002884775_UMB1686         TIDNKFSSDSF.NKGDEFVVVEHQKKNITNTTSNLSVTSANDDRVYPGALFRADKNLMDNMPSLISANRAPITLSVD
GCA_000263595_1500E           TIDNKFSSDSF.NKGDEFVVVEHQKKNITNTTSNLSVTSANDDRVYPGALFRADKNLMDNMPSLISANRAPITLSVD
GCA_000414445_JCP8481B        TIDNKFSSDSF.NNGSEFVVVEHQKKNITNTTSNLSVTSANDDRVYPGALFRADKNLMDNMPSLISANRAPITLSVD
GCA_000414465_JCP8481A        TIDNKFSSDSF.NNGSEFVVVEHQKKNITNTTSNLSVTSANDDRVYPGALFRADKNLMDNMPSLISANRAPITLSVD
GCA_001660735_23-12           TIDNKFSSDSF.NKGDEFVVVEHQKKNITNTTSNLSVTSANDDRVYPGALFRADQNLMDNMPSLISGNRAPITLSVD
GCA_003408845_NR010           TIDNKFSSDSF.DKGGEFVVVEHQKKNITNTTSNLSVTSANNDRVYPGALFRADQNLMDNMPSLISANRAPITLSVD
GCA_003426445_NR037           XXXXXXXXXXF.NKNGEFVVVEHQKKNITNTTSNLSVTSANDDRVYPGALFRADKNLMDNMPSLISANRAPITLSVD
GCA_002896555_KA00225         SIDNKFSNDSF.DKGGEFVVVEHQKKNITNTTSNLSVTSSNDARVYPGALFRADQNLMDNVPTLISANRGPLTLSVD
GCA_001546485_PSS_7772B       TIDNKFSSDSF.NNGSEFVVVEHQKKNITNTTSNLSVTSANDDRVYPGALFRADKNLMDNMPSLISANRAPITLSVD
GCA_000025205_409-05          TINNKFSSDSF.NNNGEFVVVEHQKKNITNTTSNLSVTSANNDRVYPGALFRADQNLMDNMPSLISANRAPLTLSID
GCA_000176495_5-1             TINNKFSSDSF.NKNGEFVVVEHQKKNITNTTSNLSVTSANNDRVYPGALFRADQNLMDNMPSLISANRAPLTLSID
GCA_001953155_GV37            TINNKFSSDSF.NNNGEFVVVEHQKKNITNTTSNLSVTSANNDRVYPGALFRADQNLMDNMPSLISANRAPLTLSID
GCA_002884795_UMB1642         TINNKFSSDSF.NKNGEFVVVEHQKKNITNTTSNLSVTSANNDRVYPGALFRADQNLMDNMPSLISANRAPLTLSID
GCA_002884855_UMB0170         TINNKFSSDSF.NNNGEFVVVEHQKKNITNTTSNLSVTSANNDRVYPGALFRADQNLMDNMPSLISANRAPLTLSID
GCA_002884875_UMB0264         TINNKFSSDSF.NNNGEFVVVEHQKKNITNTTSNLSVTSANNDRVYPGALFRADQNLMDNMPSLISANRAPLTLSID
GCA_003397705_GS9838-1        TINNKFSSDSF.NNNGEFVVVEHQKKNITNTTSNLSVTSANNDRVYPGALFRADQNLMDNMPSLISANRAPLTLSID
GCA_003397745_GS10234         TINNKFSSDSF.NNNGEFVVVEHQKKNITNTTSNLSVTSANNDRVYPGALFRADQNLMDNMPSLISANRAPLTLSID
GCA_003408815_N72             TINNKFSSDSF.NKNGEFVVVEHQKKNITNTTSNLSVTSANNDRVYPGALFRADQNLMDNMPSLISANRAPLTLSID
GCA_002861125_UMB0912         TINNKFSSDSF.NKNGEFVVVEHQKKNITNTTSNLSVTSANNDRVYPGALFRADQNLMDNMPSLISANRAPLTLSID
GCA_002861145_UMB0913         TINNKFSSDSF.NKNGEFVVVEHQKKNITNTTSNLSVTSANNDRVYPGALFRADQNLMDNMPSLISANRAPLTLSID
GCA_003397635_UGent09.48      TINNKFSSDSF.NKNGEFVVVEHQKKNITNTTSNLSVTSANNDRVYPGALFRADQNLMDNMPSLISANRAPLTLSID
GCA_000263575_6420B           TINNKFSSDSF.NKNGEFVVVEHQKKNITNTTSNLSVTSANNDRVYPGALFRADQNLMDNMPSLISANRAPLTLSID
GCA_000176475_AMD             TINNKFSSDSF.NKNGEFVVVEHQKKNITNTTSNLSVTSANNDRVYPGALFRADQNLMDNMPSLISANRAPLTLSID
GCA_003293675_UGent06.41      TINNKFSSDSF.NKNGEFVVVEHQKKNITNTTSNLSVTSANNDRVYPGALFRADQNLMDNMPSLISANRAPLTLSID
GCA_002862065_UMB0682         TINNKFSSDSF.NKNGEFVVVEHQKKNITNTTSNLSVTSANNDRVYPGALFRADQNLMDNMPSLISANRAPLTLSID
GCA_001563665_CMW7778B        SIDNKFSSDSF.DKGGEFIVVEHQKKNITNTTSNLSVTSANNARVYPGALFRADQNLMDNVPTLISANRGPLTLSVD
```

```
                               150       160       170       180       190       200       210       220
WP_009567818.1_Sintermedius    IPVNRGKTTISVNLPGLKNGESNLTVENPSNSTVRTAVNNLVEKWIQKYSKTHAVPARMQYESISAQSMSQLQAKFG
WP_006730404.1_Liners          LPGMTDNTNAKLVAHPTTSSVNSAVNDLVEKWIAKDSVNHAIPARIEYDTTSAQSMDQLKVKFGADFAKISVPLKID
GCA_003397685_ATCC14018        LPGFHGGESAVTVQRPTKSSVTSAVNGLVSKWNAQYGASHHVAARMQYDSASAQSMNQLKAKFGADFAKIGVPLKID
GCA_000159155_ATCC14019        LPGFHGGESAVTVQRPTKSSVTSAVNGLVSKWNAQYGASHHVAARMQYDSASAQSMNQLKAKFGADFAKIGVPLKID
GCA_900637625_NCTC10287        LPGFHGGESAVTVQRPTKSSVTSAVNGLVSKWNAQYGASHHVAARMQYDSASAQSMNQLKAKFGADFAKIGVPLKID
GCA_001042655_JCM11026         LPGFHGGESAVTVQRPTKSSVTSAVNGLVSKWNAQYGASHHVAARMQYDSASAQSMNQLKAKFGADFAKIGVPLKID
GCA_900105405_DSM4944          LPGFHGGESAVTVQRPTKSSVTSAVNGLVSKWNAQYGASHHVAARMQYDSASAQSMNQLKAKFGADFAKIGVPLKID
GCA_003034925_ATCC49145        LPGFHGGESAVTVQRPTKSSVTSAVNGLVSKWNAQYGASHHVAARMQYDSASAQSMNQLKAKFGADFAKIGVPLKID
GCA_003812765_FDAARGOS_568     LPGFHGGESAVTVQRPTKSSVTSAVNGLVSKWNAQYGASHHVAARMQYDSASAQSMNQLKAKFGADFAKIGVPLKID
GCA_002206225_FDAARGOS_296     LPGFHGGESAVTVQRPTKSSVTSAVNGLVSKWNAQYGASHHVAARMQYDSASAQSMNQLKAKFGADFAKIGVPLKID
GCA_002861975_UMB0298          LPGFHGGESAVTVQRPTKSSVTSAVNGLVSKWNAQYGASHHVAARMQYDSASAQSMNQLKAKFGADFAKIGVPLKID
GCA_002861965_UMB0386          LPGFHGGESAVTVQRPTKSSVTSAVNGLVSKWNAQYGASHHVAARMQYDSASAQSMNQLKAKFGADFAKIGVPLKID
GCA_003397605_UGent25.49       LPGFHGGESAVTVQRPTKSSVTSTVNGLVSKWNAQYAASHHVAARMQYDSASAQSMNQLKAKFGADFAKIGVPLKID
GCA_003408745_GH015            LPGFHGGESAVTVQRPTKSSVTSAVNGLVSKWNAQYGASHHVAARMQYDSASAQSMNQLKAKFGADFAKIGVPLKID
GCA_002861945_UMB0770          LPGFHGGESAVTVQRPTKSSVTSAVNGLVSKWNAQYGASHHVAARMQYDSASAQSMNQLKAKFGADFAKIGVPLKID
GCA_001049785_3549624          LPGFHGGESAVTVQRPTKSSVTSAVNGLVSKWNAQYGASHHVAARMQYDSASAQSMNQLKAKFGADFAKIGVPLKID
GCA_000213955_HMP9231          LPGFHGGESAVTVQRPTKSSVTSAVNGLVSKWNAQYGASHHVAARMQYDSASAQSMNQLKAKFGADFAKIGVPLKID
GCA_002861925_UMB0775          LPGFHGGESAVTVQRPTKSSVTSAVNGLVSKWNAQYGASHHVAARMQYDSASAQSMNQLKAKFGADFAKIGVPLKID
GCA_003426545_NR001            LPGFHGGESAVTVKHPTKSSVTSAVNGLVSKWNAQYGASHHVAARMQYDSASAQSMNQLKAKFGADFAKIGVPLKID
GCA_003585655_NR038            LPGFHGGESAVTVKHPTKSSVTSAVNGLVSKWNAQYGASHHVAARMQYDSASAQSMNQLKAKFGADFAKIGVPLKID
GCA_002894105_DNF01149         LPGFHGGESAVTVQRPTKSSVTSAVNGLVSKWNAQYGASHHVAARMQYDSASAQSMNQLKAKFGADFAKIGVPLKID
GCA_000263435_284V             LPGFHGGESAVTVQRPTKSSVTSAVNGLVSKWNAQYGASHHVAARMQYDSASAQSMNQLKAKFGADFAKIGVPLKID
GCA_003426285_WP023            LPGFHGGESAVTVQRPTKSSVTSAVNGLVSKWNAQYGASHHVAARMQYDSASAQSMNQLKAKFGADFAKIGVPLKID
GCA_002884835_UMB0768          LPGFHGGESAVTVQRPTKSSVTSAVNGLVSKWNAQYGASHHVAARMQYDSASAQSMNQLKAKFGADFAKIGVPLKID
GCA_003397665_UGent09.07       LPGFHGGESAVTVQRPTKSSVTSAVNGLVSKWNAQYGASHHVAARMQYDSASAQSMNQLKAKFGADFAKIGVPLKID
GCA_000263555_0288E            LPGFHGGESAVTVKHPTKSSVTSAVNGLVSKWNAQYGASHHVAARMQYDSASAQSMNQLKAKFGADFAKIGVPLKID
GCA_000414685_JCP7276          LPGFHGGESAVTVKHPTKSSVTSAVNGLVSKWNAQYGASHHVAARMQYDSASAQSMNQLKAKFGADFAKIGVPLKID
GCA_000414645_JCP7672          LPGFHGGESAVTVKHPTKSSVTSAVNGLVSKWNAQYGASHHVAARMQYDSASAQSMNQLKAKFGADFAKIGVPLKID
GCA_003397755_UGent09.01       LPGFHGGESAVTVQRPTKSSVTSTVNGLVSKWNAQYAASHHVAARMQYDSASAQSMNQLKAKFGADFAKIGVPLKID
GCA_000263515_00703C2mash      LPGFHGGESAVTVQRPTKSSVTSAVNGLVSKWNAQYAASHHVAARMQYDSASAQSMNQLKAKFGADFAKIGVPLKID
GCA_000263615_00703Bmash       LPGFHGGESAVTVQRPTKSSVTSAVNGLVSKWNAQYAASHHVAARMQYDSASAQSMNQLKAKFGADFAKIGVPLKID
GCA_003408835_N144             LPGFHGGESAVTVQRPTKSSVTSAVNGLVSKWNAQYAASHHVAARMQYDSASAQSMNQLKAKFGADFAKIGVPLKID
GCA_001546445_GED7275B         LPGFHGGESAVTVEHPTKSSVTSAVNGLVSKWNAQYAASHHVAARMQYDSASAQSMNQLKAKFGADFAKIGVPLKID
GCA_000414605_JCP8017A         LPGFHGGESAVTVQRPTKSSVTSAVNGLVSKWNAQYAASHHVAARMQYDSASAQSMNQLKAKFGADFAKIGVPLKID
GCA_000414585_JCP8017B         LPGFHGGESAVTVQRPTKSSVTSAVNGLVSKWNAQYAASHHVAARMQYDSASAQSMNQLKAKFGADFAKIGVPLKID
GCA_000414665_JCP7659          LPGFHGGESAVTVQRPTKSSVTSAVNGLVSKWNAQYAASHHVAARMQYDSASAQSMNQLKAKFGADFAKIGVPLKID
GCA_003408775_N160             LPGFHGGESAVTVQRPTKSSVTSAVNGLVSKWNAQYAASHHVAARMQYDSASAQSMNQLKAKFGADFAKIGVPLKID
GCA_000263655_6119V5           LPGFHGGESAVTVQRPTKSSVTSAVNGLVSKWNAQYAASHHVAARMQYDSASAQSMNQLKAKFGADFAKIGVPLKID
GCA_000263535_75712            LPGFHGGESAVTVQRPTKSSVTSAVNGLVSKWNAQYGASHHVAARMQYDSASAQSMNQLKAKFGADFAKIGVPLKID
GCA_003585755_NR039            LPGFHGGESAVTVQRPTKSSVTSAVNGLVSKWNAQYGASHHVAARMQYDSASAQSMNQLKAKFGADFAKIGVPLKID
GCA_002862045_UMB0233          LPGFHGGESAVTVQRPTKSSVTSTVNGLVSKWNAQYAASHHVAARMQYDSASAQSMNQLKAKFGADFAKIGVPLKID
GCA_000214315_315-A            LPGFHGGESAVTVQRPTKSSVTSTVNGLVSKWNAQYAASHHVAARMQYDSASAQSMNQLKAKFGADFAKIGVPLKID
GCA_002862005_UMB0032B         LPGFHGGESAVTVQRPTKSSVTSTVNGLVSKWNAQYAASHHVAARMQYDSASAQSMNQLKAKFGADFAKIGVPLKID
GCA_002862015_UMB0032A         LPGFHGGESAVTVQRPTKSSVTSTVNGLVSKWNAQYAASHHVAARMQYDSASAQSMNQLKAKFGADFAKIGVPLKID
GCA_002861165_UMB0061          LPGFHGGESAVTVQRPTKSSVTSAVNGLVSKWNAQYGASHHVAARMQYDSASAQSMNQLKAKFGADFAKIGVPLKID
GCA_001660755_18-4             LPGFHGGESAVTVQRPTKSSVTSAVNGLVSKWNAQYGASHHVAARMQYDSASAQSMNQLKAKFGADFAKIGVPLKID
GCA_000165635_41V              LPGFHGGESAVTVQRPTKSSVTSAVNSLVSKWNAQYAASHHVAARMQYDSASAQSMNQLKAKFGADFAKIGVPLKID
GCA_000263475_55152            LPGFHGGESAVTVQRPTKSSVTSAVNSLVSKWNAQYAASHHVAARMQYDSASAQSMNQLKAKFGADFAKIGVPLKID
GCA_000263495_1400E            LPGFHGGESAVTVQRPTKSSVTSAVNSLVSKWNAQYAASHHVAARMQYDSASAQSMNQLKAKFGADFAKIGVPLKID
GCA_000414525_JCP8108          LPGFHGGESAVTVQRPTKSSVTSAVNSLVSKWNAQYAASHHVAARMQYDSASAQSMNQLKAKFGADFAKIGVPLKID
GCA_002861905_UMB0830          LPGFHGGESAVTVQRPTKSSVTSTVNGLVSKWNAQYAASHHVAARMQYDSASAQSMNQLKAKFGADFAKIGVPLKID
GCA_003369895_N101             LPGFHGGESAVTVQRPTKSSVTSTVNGLVSKWNAQYAASHHVAARMQYDSASAQSMNQLKAKFGADFAKIGVPLKID
GCA_003369935_N153             LPGFHGGESAVTVQRPTKSSVTSTLNGLVSKWNAQYAASHHVAARMQYDSASAQSMNQLKAKFGADFAKIGVPLKID
GCA_003369965_N95              LPGFHGGESAVTVQRPTKSSVTSTVNGLVSKWNAQYAASHHVAARMQYDSASAQSMNQLKAKFGADFAKIGVPLKID
GCA_000414625_JCP7719          LPGFHGGESAVTVQRPTKSSVTSTVNGLVSKWNAQYAASHHVAARMQYDSASAQSMNQLKAKFGADFAKIGVPLKID
GCA_000414485_JCP8151B         LPGFHGGESAVTVQRPTKSSVTSTVNGLVSKWNAQYAASHHVAARMQYDSASAQSMNQLKAKFGADFAKIGVPLKID
GCA_000263635_00703Dmash       LPGFHGGESAVTVQRPTKSSVTSAVNGLVSKWNAQYAASHHVAARMQYDSASAQSMNQLKAKFGADFAKIGVPLKID
GCA_000165615_101              LPGFHGGESAVTVQRPTKSSVTSAVNGLVSKWNAQYAASHHVAARMQYDSASAQSMNQLKAKFGADFAKIGVPLKID
GCA_002884775_UMB1686          LPGFHGGESAVTVQRPTKSSVTSAVNGLVSKWNAQYAASHHVAARMQYDSASAQSMNQLKAKFGADFAKIGVPLKID
GCA_000263595_1500E            LPGFHGGESAVTVQRPTKSSVTSAVNGLVSKWNAQYAASHHVAARMQYDSASAQSMNQLKAKFGADFAKIGVPLKID
GCA_000414445_JCP8481B         LPGFHGGESAVTVQRPTKSSVTSAVNGLVSKWNAQYAASHHVAARMQYDSASAQSMNQLKAKFGADFAKIGVPLKID
GCA_000414465_JCP8481A         LPGFHGGESAVTVQRPTKSSVTSAVNGLVSKWNAQYAASHHVAARMQYDSASAQSMNQLKAKFGADFAKIGVPLKID
GCA_001660735_23-12            LPGFHGGESAVTVQRPTKSSVTSTVNGLVSKWNAQYAASHHVAARMQYDSASAQSMNQLKAKFGADFAKIGVPLKID
GCA_003408845_NR010            LPGFHGGESAVTVQRPTKSSVTSAVNSLVSKWNAQYAASHHVAARMQYDSASAQSMNQLKAKFGADFAKIGVPLKID
GCA_003426445_NR037            LPGFHGGESAVTVQRPTKSSVTSAVNGLVSKWNAQYGASHHVAARMQYDSASAQSMNQLKAKFGADFAKIGVPLKID
GCA_002896555_KA00225          LPGFHNGESVATVTRPTKSTVTSAVNDLVSKWNSQYAATHHVAAKMQYDSASAQSMNQLKAKFGADFAKIGVPLKID
GCA_001546485_PSS_7772B        LPGFHGGESAVTVQRPTKSSVTSAVNGLVSKWNAQYAASHHVAARMQYDSASAQSMNQLKAKFGADFAKIGVPLKID
GCA_000025205_409-05           LPGFHGGESAVTVQHPTKSSVTSAVNGLVSKWNAQYAASHHVAARMQYDSASAQSMNQLKAKFGADFAKIGVPLKID
GCA_000176495_5-1              LPGFHGGESAVTVQHPTKSSVTSAVNGLVSKWNAQYAASHHVAARMQYDSASAQSMNQLKAKFGADFAKIGVPLKID
GCA_001953155_GV37             LPGFHGGESAVTVQHPTKSSVTSAVNGLVSKWNAQYAASHHVAARMQYDSASAQSMNQLKAKFGADFAKIGVPLKID
GCA_002884795_UMB1642          LPGFHGGESAVTVQHPTKSSVTSAVNGLVSKWNAQYAASHHVAARMQYDSASAQSMNQLKAKFGADFAKIGVPLKID
GCA_002884855_UMB0170          LPGFHGGESAVTVQHPTKSSVTSAVNGLVSKWNAQYAASHHVAARMQYDSASAQSMNQLKAKFGADFAKIGVPLKID
GCA_002884875_UMB0264          LPGFHGGESAVTVQHPTKSSVTSAVNGLVSKWNAQYAASHHVAARMQYDSASAQSMNQLKAKFGADFAKIGVPLKID
GCA_003397705_GS9838-1         LPGFHGGESAVTVQHPTKSSVTSAVNGLVSKWNAQYAASHHVAARMQYDSASAQSMNQLKAKFGADFAKIGVPLKID
GCA_003397745_GS10234          LPGFHGGESAVTVQHPTKSSVTSAVNGLVSKWNAQYAASHHVAARMQYDSASAQSMNQLKAKFGADFAKIGVPLKID
GCA_003408815_N72              LPGFHGGESAVTVQHPTKSSVTSAVNGLVSKWNAQYAASHHVAARMQYDSASAQSMNQLKAKFGADFAKIGVPLKID
GCA_002861125_UMB0912          LPGFHGGESAVTVQHPTKSSVTSAVNGLVSKWNAQYAASHHVAARMQYDSASAQSMNQLKAKFGADFAKIGVPLKID
GCA_002861145_UMB0913          LPGFHGGESAVTVQHPTKSSVTSAVNGLVSKWNAQYAASHHVAARMQYDSASAQSMNQLKAKFGADFAKIGVPLKID
GCA_003397635_UGent09.48       LPGFHGGESAVTVQHPTKSSVTSAVNGLVSKWNAQYAASHHVAARMQYDSASAQSMNQLKAKFGADFAKIGVPLKID
GCA_000263575_6420B            LPGFHGGESAVTVQHPTKSSVTSAVNGLVSKWNAQYAASHHVAARMQYDSASAQSMNQLKAKFGADFAKIGVPLKID
GCA_000176475_AMD              LPGFHGGESAVTVQHPTKSSVTSAVNGLVSKWNAQYAASHHVAARMQYDSASAQSMNQLKAKFGADFAKIGVPLKID
GCA_003293675_UGent06.41       LPGFHGGESAVTVQHPTKSSVTSAVNGLVSKWNAQYAASHHVAARMQYDSASAQSMNQLKAKFGADFAKIGVPLKID
GCA_002862065_UMB0682          LPGFHGGESAVTVQHPTKSSVTSAVNGLVSKWNAQYAASHHVAARMQYDSASAQSMNQLKAKFGADFAKIGVPLKID
GCA_001563665_CMW7778B         LPGFHDGESVATVKRPTKSTVTSAVNDLVYKWNAQYAASHQVAAKMQYDSASAQSMNQLKAKFGADFAKIGVPLKID
```

```
                              230       240       250       260       270       280       290       300
WP_009567818.1_Sintermedius   ADFSKVGAPLNVDFSSVHKGEKQVFIANFRQVYYTASVDSPNSPSALFGSGITPTDLINRGVNSKTPPVYVSNVSYG
WP_006730404.1_Liners         FDAIHNGEKQASIVNFKQIYYTASVDAPENPGDVFDAHVTAKDLQKRGINSKTPLVYVSSVSYGRSMYIKLETTSKS
GCA_003397685_ATCC14018       FDAVHKGEKQTQIVNFKQTYYTVSVDAPDSPADFFAPCTTPDSLKNRGVDNKRPPVYVSNVAYGRSMYVKFDTTSKS
GCA_000159155_ATCC14019       FDAVHKGEKQTQIVNFKQTYYTVSVDAPDSPADFFAPCTTPDSLKNRGVDNKRPPVYVSNVAYGRSMYVKFDTTSKS
GCA_900637625_NCTC10287       FDAVHKGEKQTQIVNFKQTYYTVSVDAPDSPADFFAPCTTPDSLKNRGVDNKRPPVYVSNVAYGRSMYVKFDTTSKS
GCA_001042655_JCM11026        FDAVHKGEKQTQIVNFKQTYYTVSVDAPDSPADFFAPCTTPDSLKNRGVDNKRPPVYVSNVAYGRSMYVKFDTTSKS
GCA_900105405_DSM4944         FDAVHKGEKQTQIVNFKQTYYTVSVDAPDSPADFFAPCTTPDSLKNRGVDNKRPPVYVSNVAYGRSMYVKFDTTSKS
GCA_003034925_ATCC49145       FDAVHKGEKQTQIVNFKQTYYTVSVDAPDSPADFFAPCTTPDSLKNRGVDNKRPPVYVSNVAYGRSMYVKFDTTSKS
GCA_003812765_FDAARGOS_568    FDAVHKGEKQTQIVNFKQTYYTVSVDAPDSPADFFAPCTTPDSLKNRGVDNKRPPVYVSNVAYGRSMYVKFDTTSKS
GCA_002206225_FDAARGOS_296    FDAVHKGEKQTQIVNFKQTYYTVSVDAPDSPADFFAPCTTPDSLKNRGVDNKRPPVYVSNVAYGRSMYVKFDTTSKS
GCA_002861975_UMB0298         FDAVHKGEKQTQIVNFKQTYYTVSVDAPDSPADFFAPCTTPDSLKNRGVDNKRPPVYVSNVAYGRSMYVKFDTTSKS
GCA_002861965_UMB0386         FDAVHKGEKQTQIVNFKQTYYTVSVDAPDSPADFFAPCTTPDSLKNRGVDNKRPPVYVSNVAYGRSMYVKFDTTSKS
GCA_003397605_UGent25.49      FDAVHKGEKQTQIVNFKQTYYTVSVDAPDSPADFFAPCTTPESLKSRGVDSKRPPVYVSNVAYGRSMYVKFDTRSKS
GCA_003408745_GH015           FDAVHKGEKQTQIVNFKQTYYTVSVDAPDSPADFFAPCTTPDSLKNRGVDNKRPPVYVSNVAYGRSMYVKFDTTSKS
GCA_002861945_UMB0770         FDAVHKGEKQTQIVNFKQTYYTVSVDAPDSPADFFAPCTTPDSLKNRGVDNKRPPVYVSNVAYGRSMYVKFDTTSKS
GCA_001049785_3549624         FDAVHKGEKQTQIVNFKQTYYTVSVDAPDSPADFFAPCTTPDSLKNRGVDNKRPPVYVSNVAYGRSMYVKFDTTSKS
GCA_000213955_HMP9231         FDAVHKGEKQTQIVNFKQTYYTVSVDAPDSPADFFAPCTTPDSLKNRGVDNKRPPVYVSNVAYGRSMYVKFDTTSKS
GCA_002861925_UMB0775         FDAVHKGEKQTQIVNFKQTYYTVSVDAPDSPADFFAPCTTPDSLKNRGVDNKRPPVYVSNVAYGRSMYVKFDTTSKS
GCA_003426545_NR001           FDAVHKGEKQTQIVNFKQTYYTVSVDAPDSPADFFAPCTTPDSLKNRGVDNKRPPVYVSNVAYGRSMYVKFDTTSKS
GCA_003585655_NR038           FDAVHKGEKQTQIVNFKQTYYTVSVDAPDSPADFFAPCTTPDSLKNRGVDNKRPPVYVSNVAYGRSMYVKFDTTSKS
GCA_002894105_DNF01149        FDAVHKGEKQTQIVNFKQTYYTVSVDAPDSPADFFAPCTTPDSLKNRGVDNKRPPVYVSNVAYGRSMYVKFDTTSKS
GCA_000263435_284V            FDAVHKGEKQTQIVNFKQTYYTVSVDAPDSPADFFAPCTTPDSLKNRGVDNKRPPVYVSNVAYGRSMYVKFDTTSKS
GCA_003426285_WP023           FDAVHKGEKQTQIVNFKQTYYTVSVDAPDSPADFFAPCTTPDSLKNRGVDNKRPPVYVSNVAYGRSMYVKFDTTSKS
GCA_002884835_UMB0768         FDAVHKGEKQTQIVNFKQTYYTVSVDAPDSPADFFAPCTTPDSLKNRGVDNKRPPVYVSNVAYGRSMYVKFDTTSKS
GCA_003397665_UGent09.07      FDAVHKGEKQTQIVNFKQTYYTVSVDAPDSPADFFAPCTTPDSLKNRGVDNKRPPVYVSNVAYGRSMYVKFDTTSKS
GCA_000263555_0288E           FDAVHKGEKQTQIVSFKQTYYTVSVDAPDSPADFFAPCTTPDSLKNRGVDNKRPPVYVSNVAYGRSMYVKFDTTSKS
GCA_000414685_JCP7276         FDAVHKGEKQTQIVNFKQTYYTVSVDAPDSPADFFAPCTTPDSLKNRGVDNKRPPVYVSNVAYGRSMYVKFDTTSKS
GCA_000414645_JCP7672         FDAVHKGEKQTQIVNFKQTYYTVSVDAPDSPADFFAPCTTPDSLKNRGVDNKRPPVYVSNVAYGRSMYVKFDTTSKS
GCA_003397755_UGent09.01      FDAVHKGEKQTQIVNFKQTYYTVSVDAPDSPADFFAPCTTPESLKSRGVDSKRPPVYVSNVAYGRSMYVKFDTRSKS
GCA_000263515_00703C2mash     FDAVHKGEKQTQIVNFKQTYYTVSVDAPDSPADFFAPCTTPESLKNRGVDNKRPPVYVSNVAYGRSMYVKFDTHSKS
GCA_000263615_00703Bmash      FDAVHKGEKQTQIVNFKQTYYTVSVDAPDSPADFFAPCTTPESLKSRGVDSKRPPVYVSNVAYGRSMYVKFDTRSKS
GCA_003408835_N144            FDAVHKGEKQTQIVNFKQTYYTVSVDAPDSPADFFAPCTTPESLKNRGVDNKRPPVYVSNVAYGRSMYVKFDTHSKS
GCA_001546445_GED7275B        FDAVHKGEKQTQIVNFKQTYYTVSVDAPDSPADFFAPCTTPESLKSRGVDSKRPPVYVSNVAYGRSMYVKFDTRSKS
GCA_000414605_JCP8017A        FDAVHKGEKQTQIVNFKQTYYTVSVDAPDSPADFFAPCTTPESLKSRGVDSKRPPVYVSNVAYGRSMYVKFDTRSKS
GCA_000414585_JCP8017B        FDAVHKGEKQTQIVNFKQTYYTVSVDAPDSPADFFAPCTTPESLKSRGVDSKRPPVYVSNVAYGRSMYVKFDTRSKS
GCA_000414665_JCP7659         FDAVHKGEKQTQIVNFKQTYYTVSVDAPDSPADFFAPCTTPESLKNRGVDNKRPPVYVSNVAYGRSMYVKFDTHSKS
GCA_003408775_N160            FDAVHKGEKQTQIVNFKQTYYTVSVDAPDSPADFFAPCTTPESLKNRGVDSQRPPVYVSNVAYGRSMYVKFDTRSKS
GCA_000263655_6119V5          FDAVHKGEKQTQIVNFKQTYYTVSVDAPDSPADFFAPCTTPESLKNRGVDSQRPPVYVSNVAYGRSMYVKFDTRSKS
GCA_000263535_75712           FDAVHKGEKQTQIVNFKQTYYTVSVDAPDSPADFFAPCTTPDSLKNRGVDNKRPPVYVSNVAYGRSMYVKFDTTSKS
GCA_003585755_NR039           FDAVHKGEKQTQIVNFKQTYYTVSVDAPDSPADFFAPCTTPDSLKNRGVDNKRPPVYVSNVAYGRSMYVKFDTTSKS
GCA_002862045_UMB0233         FDAVHKGEKQTQIVNFKQTYYTVSVDAPDSPADFFAPCTTPESLKSRGVDSKRPPVYVSNVAYGRSMYVKFDTRSKS
GCA_000214315_315-A           FDAVHKGEKQTQIVNFKQTYYTVSVDAPDSPADFFAPCTTPESLKSRGVDSKRPPVYVSNVAYGRSMYVKFDTRSKS
GCA_002862005_UMB0032B        FDAVHKGEKQTQIVNFKQTYYTVSVDAPDSPADFFAPCTTPESLKSRGVDSKRPPVYVSNVAYGRSMYVKFDTRSKS
GCA_002862015_UMB0032A        FDAVHKGEKQTQIVNFKQTYYTVSVDAPDSPADFFAPCTTPESLKSRGVDSKRPPVYVSNVAYGRSMYVKFDTRSKS
GCA_002861165_UMB0061         FDAVHKGEKQTQIVNFKQTYYTVSVDAPDSPADFFAPCTTPDSLKNRGVDNKRPPVYVSNVAYGRSMYVKFDTTSKS
GCA_001660755_18-4            FDAVHKGEKQTQIVNFKQTYYTVSVDAPDSPADFFAPCTTPDSLKNRGVDNKRPPVYVSNVAYGRSMYVKFDTTSKS
GCA_000165635_41V             FDAVHKGEKQTQIVNFKQTYYTVSVDAPDSPADFFAPCTTPENLKNRGVDSKRPPVYVSNVAYGRSMYVKFDTRSKS
GCA_000263475_55152           FDAVHKGEKQTQIVNFKQTYYTVSVDAPDSPADFFAPCTTPENLKNRGVDSKRPPVYVSNVAYGRSMYVKFDTRSKS
GCA_000263495_1400E           FDAVHKGEKQTQIVNFKQTYYTVSVDAPDSPADFFAPCTTPENLKNRGVDSKRPPVYVSNVAYGRSMYVKFDTRSKS
GCA_000414525_JCP8108         FDAVHKGEKQTQIVNFKQTYYTVSVDAPDSPADFFAPCTTPENLKNRGVDSKRPPVYVSNVAYGRSMYVKFDTRSKS
GCA_002861905_UMB0830         FDAVHKGEKQTQIVNFKQTYYTVSVDAPDSPADFFAPCTTPESLKSRGVDSKRPPVYVSNVAYGRSMYVKFDTRSKS
GCA_003369895_N101            FDAVHKGEKQTQIVNFKQTYYTVSVDAPDSPADFFAPCTTPESLKSRGVDSKRPPVYVSNVAYGRSMYVKFDTRSKS
GCA_003369935_N153            FDAVHKGEKQTQIVNFKQTYYTVSVDAPDSPADFFAPCTTPESLKSRGVDSKRPPVYVSNVAYGRSMYVKFDTRSKS
GCA_003369965_N95             FDAVHKGEKQTQIVNFKQTYYTVSVDAPDSPADFFAPCTTPESLKSRGVDSKRPPVYVSNVAYGRSMYVKFDTRSKS
GCA_000414625_JCP7719         FDAVHKGEKQTQIVNFKQTYYTVSVDAPDSPADFFAPCTTPESLKSRGVDSKRPPVYVSNVAYGRSMYVKFDTRSKS
GCA_000414485_JCP8151B        FDAVHKGEKQTQIVNFKQTYYTVSVDAPDSPADFFAPCTTPESLKSRGVDSKRPPVYVSNVAYGRSMYVKFDTRSKS
GCA_000263635_00703Dmash      FDAVHKGEKQTQIVNFKQTYYTVSVDAPDSPADFFAPCTTPESLKSRGVDSKRPPVYVSNVAYGRSMYVKFDTRSKS
GCA_000165615_101             FDAVHKGEKQTQIVNFKQTYYTVSVDAPDSPADFFAPCTTPESLKSRGVDSKRPPVYVSNVAYGRSMYVKFDTRSKS
GCA_002884775_UMB1686         FDAVHKGEKQTQIVNFKQTYYTVSVDAPDSPADFFAPCTTPESLKSRGVDSKRPPVYVSNVAYGRSMYVKFDTRSKS
GCA_000263595_1500E           FDAVHKGEKQTQIVNFKQTYYTVSVDAPDSPADFFAPCTTPESLKSRGVDSKRPPVYVSNVAYGRSMYVKFDTRSKS
GCA_000414445_JCP8481B        FDAVHKGEKQTQIVNFKQTYYTVSVDAPDSPADFFAPCTTAENLKSRGIDSKRPPVYVSNVSYGRSMYVKFDTRSKS
GCA_000414465_JCP8481A        FDAVHKGEKQTQIVNFKQTYYTVSVDAPDSPADFFAPCTTAENLKSRGIDSKRPPVYVSNVSYGRSMYVKFDTRSKS
GCA_001660735_23-12           FDAVHKGEKQTQIVNFKQTYYTVSVDAPDSPADFFAPCTTPESLKSRGVDSKRPPVYVSNVAYGRSMYVKFDTRSKS
GCA_003408845_NR010           FDAVHKGEKQTQIVNFKQTYYTVSVDAPDSPADFFAPCTTPESLKNRGIDSKRPPVYVSNVAYGRSMYVKFDTRSKS
GCA_003426445_NR037           FDAVHKGXXXXXXXXXXXXXXXXXXXXXDSPADFFAPCTTPDSLKNRGVDNKRPPVYVSNVAYGRSMYVKFDTTSKS
GCA_002896555_KA00225         FDAVHRGEKQTQIVNFKQTYYNVSVDAPDSPSDFFAPCTSPESLKRRGVDEKHPPVYVSNVAYGRSMYVKFDTNSKS
GCA_001546485_PSS_7772B       FDAVHKGEKQTQIVNFKQTYYTVSVDAPDSPADFFAPCTTAENLKSRGIDSKRPPVYVSNVSYGRSMYVKFDTRSKS
GCA_000025205_409-05          FDAVHKGEKQTQIVNFKQTYYTVSVDAPDSPADFFAPCTTPESLKNRGVDSKRPPVYVSNVAYGRSMYVKFDTTSKS
GCA_000176495_5-1             FDAVHKGEKQTQIVNFKQTYYTVSVDAPDSPADFFAPCTTPESLKNRGVDSKRPPVYVSNVAYGRSMYVKFDTTSKS
GCA_001953155_GV37            FDAVHKGEKQTQIVNFKQTYYTVSVDAPDSPADFFAPCTTPESLKNRGVDSKRPPVYVSNVAYGRSMYVKFDTTSKS
GCA_002884795_UMB1642         FDAVHKGEKQTQIVNFKQTYYTVSVDAPDSPADFFAPCTTPESLKNRGVDSKRPPVYVSNVAYGRSMYVKFDTTSKS
GCA_002884855_UMB0170         FDAVHKGEKQTQIVNFKQTYYTVSVDAPDSPADFFAPCTTPESLKNRGVDSKRPPVYVSNVAYGRSMYVKFDTTSKS
GCA_002884875_UMB0264         FDAVHKGEKQTQIVNFKQTYYTVSVDAPDSPADFFAPCTTPESLKNRGVDSKRPPVYVSNVAYGRSMYVKFDTTSKS
GCA_003397705_GS9838-1        FDAVHKGEKQTQIVNFKQTYYTVSVDAPDSPADFFAPCTTPESLKNRGVDSKRPPVYVSNVAYGRSMYVKFDTTSKS
GCA_003397745_GS10234         FDAVHKGEKQTQIVNFKQTYYTVSVDAPDSPADFFAPCTTPESLKNRGVDSKRPPVYVSNVAYGRSMYVKFDTTSKS
GCA_003408815_N72             FDAVHKGEKQTQIVNFKQTYYTVSVDAPDSPADFFAPCTTPESLKNRGVDSKRPPVYVSNVAYGRSMYVKFDTTSKS
GCA_002861125_UMB0912         FDAVHKGEKQTQIVNFKQTYYTVSVDAPDSPADFFAPCTTPESLKNRGVDSKRPPVYVSNVAYGRSMYVKFDTTSKS
GCA_002861145_UMB0913         FDAVHKGEKQTQIVNFKQTYYTVSVDAPDSPADFFAPCTTPESLKNRGVDSKRPPVYVSNVAYGRSMYVKFDTTSKS
GCA_003397635_UGent09.48      FDAVHKGEKQTQIVNFKQTYYTVSVDAPDSPADFFAPCTTPESLKNRGVDSKRPPVYVSNVAYGRSMYVKFDTTSKS
GCA_000263575_6420B           FDAVHKGEKQTQIVNFKQTYYTVSVDAPDSPADFFAPCTTPESLKNRGVDSKRPPVYVSNVAYGRSMYVKFDTTSKS
GCA_000176475_AMD             FDAVHKGEKQTQIVNFKQTYYTVSVDAPDSPADFFAPCTTPESLKNRGVDSKRPPVYVSNVAYGRSMYVKFDTTSKS
GCA_003293675_UGent06.41      FDAVHKGEKQTQIVNFKQTYYTVSVDAPDSPADFFAPCTTPESLKNRGVDSKRPPVYVSNVAYGRSMYVKFDTTSKS
GCA_002862065_UMB0682         FDAVHKGEKQTQIVNFKQTYYTVSVDAPDSPADFFAPCTTPESLKNRGVDSKRPPVYVSNVAYGRSMYVKFDTTSKS
GCA_001563665_CMW7778B        FDAVHRGEKQTQIVNFKQTYYNVSVDAPDSPSDFFAPCTSAESLKRRGIDEKHPPVYVSNVAYGRSMYVKFDTRSKS
```

```
                                          310       320       330       340       350       360       370
                                            .         .         .         .         .         .         .
WP_009567818.1_Sintermedius   RAMYVKFETTSKSTKVQAAIDAVVKGAKLKAGTEYENILKNTKITAVVLGGNPGEASKVITGNIDTLKDLIQKGSNF
WP_006730404.1_Liners         DKVQAAFDAAIKGVKIAPNSEYDHILKNTSVVAVILGGNSGDATEVVRGDINTLKELIQKGSKFDSSNPGVAVSYGT
GCA_003397685_ATCC14018       TDFQAAVEAAIKGVEIKPNTEFHRILQNTSVTAVILGGSANGAAKVITGNIDTLKALIQEGANLSTSSPAVPIAYTT
GCA_000159155_ATCC14019       TDFQAAVEAAIKGVEIKPNTEFHRILQNTSVTAVILGGSANGAAKVITGNIDTLKALIQEGANLSTSSPAVPIAYTT
GCA_900637625_NCTC10287       TDFQAAVEAAIKGVEIKPNTEFHRILQNTSVTAVILGGSANGAAKVITGNIDTLKALIQEGANLSTSSPAVPIAYTT
GCA_001042655_JCM11026        TDFQAAVEAAIKGVEIKPNTEFHRILQNTSVTAVILGGSANGAAKVITGNIDTLKALIQEGANLSTSSPAVPIAYTT
GCA_900105405_DSM4944         TDFQAAVEAAIKGVEIKPNTEFHRILQNTSVTAVILGGSANGAAKVITGNIDTLKALIQEGANLSTSSPAVPIAYTT
GCA_003034925_ATCC49145       TDFQAAVEAAIKGVEIKPNTEFHRILQNTSVTAVILGGSANGAAKVITGNIDTLKALIQEGANLSTSSPAVPIAYTT
GCA_003812765_FDAARGOS_568    TDFQAAVEAAIKGVEIKPNTEFHRILQNTSVTAVILGGSANGAAKVITGNIDTLKALIQEGANLSTSSPAVPIAYTT
GCA_002206225_FDAARGOS_296    TDFQAAVEAAIKGVEIKPNTEFHRILQNTSVTAVILGGSANGAAKVITGNIDTLKALIQEGANLSTSSPAVPIAYTT
GCA_002861975_UMB0298         TDFQAAVEAAIKGVEIKPNTEFHRILQNTSVTAVILGGSANGAAKVITGNIDTLKALIQEGANLSTSSPAVPIAYTT
GCA_002861965_UMB0386         TDFQAAVEAAIKGVEIKPNTEFHRILQNTSVTAVILGGSANGAAKVITGNIDTLKALIQEGANLSTSSPAVPIAYTT
GCA_003397605_UGent25.49      TDFQAAVEAAIKGVEIKPNTEFHRILQNTSVTAVILGGSANGAAKVITGNIDTLKALIQEGANLSTSSPAVPIAYTT
GCA_003408745_GH015           TDFQAAVEAAIKGVEIKPNTEFHRILQNTSVTAVILGGSANGAAKVITGNIDTLKALIQEGANLSTSSPAVPIAYTT
GCA_002861945_UMB0770         TDFQAAVEAAIKGVEIKPNTEFHRILQNTSVTAVILGGSANGAAKVITGNIDTLKALIQEGANLSTSSPAVPIAYTT
GCA_001049785_3549624         TDFQAAVEAAIKGVEIKPNTEFHRILQNTSVTAVILGGSANGAAKVITGNIDTLKALIQEGANLSTSSPAVPIAYTT
GCA_000213955_HMP9231         TDFQAAVEAAIKGVEIKPNTEFHRILQNTSVTAVILGGSANGAAKVITGNIDTLKALIQEGANLSTSSPAVPIAYTT
GCA_002861925_UMB0775         TDFQAAVEAAIKGVEIKPNTEFHRILQNTSVTAVILGGSANGAAKVITGNIDTLKALIQEGANLSTSSPAVPIAYTT
GCA_003426545_NR001           TDFQAAVEAAIKGVEIKPNTEFHRILQNTSVTSVILGGSANGAAKVITGNIDTLKALIQEGANLSTSSPAVPIAYTT
GCA_003585655_NR038           TDFQAAVEAAIKGVEIKPNTEFHRILQNTSVTSVILGGSANGAAKVITGNIDTLKALIQEGANLSTSSPAVPIAYTT
GCA_002894105_DNF01149        TDFQAAVEAAIKGVEIKPNTEFHRILQNTSVTAVILGGSANGAAKVITGNIDTLKALIQEGANLSTSSPAVPIAYTT
GCA_000263435_284V            TDFQAAVEAAIKGVEIKPNTEFHRILQNTSVTAVILGGSANGAAKVITGNIDTLKALIQEGANLSTSSPAVPIAYTT
GCA_003426285_WP023           TDFQAAVEAAIKGVEIKPNTEFHRILQNTSVTAVILGGSANGAAKVITGNIDTLKALIQEGANLSTSSPAVPIAYTT
GCA_002884835_UMB0768         TDFQAAVEAAIKGVEIKPNTEFHRILQNTSVTAVILGGSANGAAKVITGNIDTLKALIQEGANLSTSSPAVPIAYTT
GCA_003397665_UGent09.07      TDFQAAVEAAIKGVEIKPNTEFHRILQNTSVTAVILGGSANGAAKVITGNIDTLKALIQEGANLSTSSPAVPIAYTT
GCA_000263555_0288E           TDFQAAVEAAIKGVEIKPNTEFHRILQNTSVTAVILGGSANGAAKVITGNIDTLKALIQEGANLSTSSPAVPIAYTT
GCA_000414685_JCP7276         TDFQAAVEAAIKGVEIKPNTEFHRILQNTSVTAVILGGSANGAAKVITGNIDTLKALIQEGANLSTSSPAVPIAYTT
GCA_000414645_JCP7672         TDFQAAVEAAIKGVEIKPNTEFHRILQNTSVTAVILGGSANGAAKVITGNIDTLKALIQEGANLSTSSPAVPIAYTT
GCA_003397755_UGent09.01      TDFQAAVEAAIKGVEIKPNTEFHRILQNTSVTAVILGGSANGAAKVITGNIDTLKALIQEGANLSTSSPAVPIAYTT
GCA_000263515_00703C2mash     TDFQAAVEAAIKGVEIKPNTEFHRILQNTSVTAVILGGSANGAAKVVTGNIDTLKSLIQEGANLSTSSPAVPIAYTT
GCA_000263615_00703Bmash      TDFQAAVEAAIKGVEIKPNTEFHRILQNTSVTAVILGGSANGAAKVVTGNIDTLKSLIQEGANLSTSSPAVPIAYTT
GCA_003408835_N144            TDFQAAVEAAIKGVEIKPNTEFHRILQNTSVTAVILGGSANGAAKVVTGNIDTLKSLIQEGANLSTSSPAVPIAYTT
GCA_001546445_GED7275B        TDFQAAVEAAIKGVEIKPNTEFHRILQNTSVTAVILGGSANGAAKVITGNVDTLKALIQEGANLSTSSPAVPIAYTT
GCA_000414605_JCP8017A        TDFQAAVEAAIKGVEIKPNTEFHRILQNTSVTAVILGGSANGAAKVITGNVDTLKALIQEGANLSTSSPAVPIAYTT
GCA_000414585_JCP8017B        TDFQAAVEAAIKGVEIKPNTEFHRILQNTSVTAVILGGSANGAAKVITGNVDTLKALIQEGANLSTSSPAVPIAYTT
GCA_000414665_JCP7659         TDFQAAVEAAIKGVEIKPNTEFHRILQNTSVTAVILGGSANGAAKVVTGNIDTLKSLIQEGANLSTSSPAVPIAYTT
GCA_003408775_N160            TDFQAAVEAAIKGVEIKPNTEFHRILQNTSVTAVILGGSANGAAKVVTGNIDTLKSLIQEGANLSTSSPAVPIAYTT
GCA_000263655_6119V5          TDFQAAVEAAIKGVEIKPNTEFHRILQNTSVTAVILGGSANGAAKVVTGNIDTLKSLIQEGANLSTSSPAVPIAYTT
GCA_000263535_75712           TDFQAAVEAAIKGVEIKPNTEFHRILQNTSVTAVILGGSANGAAKVITGNVDTLKALIQEGANLSTSSPAVPIAYTT
GCA_003585755_NR039           TDFQAAVEAAIKGVEIKPNTEFHRILQNTSVTAVILGGSANGAAKVITGNIDTLKALIQEGANLSTSSPAVPIAYTT
GCA_002862045_UMB0233         TDFQAAVEAAIKGVEIKPNTEFHRILQNTSVTAVILGGSANGAAKVITGNVDTLKALIQEGANLSTSSPAVPIAYTT
GCA_000214315_315-A           TDFQAAVEAAIKGVEIKPNTEFHRILQNTSVTAVILGGSANGAAKVITGNVDTLKALIQEGANLSTSSPAVPIAYTT
GCA_002862005_UMB0032B        TDFQAAVEAAIKGVEIKPNTEFHRILQNTSVTAVILGGSANGAAKVITGNVDTLKALIQEGANLSTSSPAVPIAYTT
GCA_002862015_UMB0032A        TDFQAAVEAAIKGVEIKPNTEFHRILQNTSVTAVILGGSANGAAKVITGNVDTLKALIQEGANLSTSSPAVPIAYTT
GCA_002861165_UMB0061         TDFQAAVEAAIKGVEIKPNTEFHRILQNTSVTAVILGGSANGAAKVITGNIDTLKALIQEGANLSTSSPAVPIAYTT
GCA_001660755_18-4            TDFQAAVEAAIKGVEIKPNTEFHRILQNTSVTAVILGGSANGAAKVITGNIDTLKALIQEGANLSTSSPAVPIAYTT
GCA_000165635_41V             TDFQAAVEAAIKGVEIKPNTEFHRILQNTSVTAVILGGSANGAAKVVTGNIDTLKALIQEGANLSTSSPAVPIAYTT
GCA_000263475_55152           TDFQAAVEAAIKGVEIKPNTEFHRILQNTSVTAVILGGSANGAAKVVTGNIDTLKALIQEGANLSTSSPAVPIAYTT
GCA_000263495_1400E           TDFQAAVEAAIKGVEIKPNTEFHRILQNTSVTAVILGGSANGAAKVVTGNIDTLKALIQEGANLSTSSPAVPIAYTT
GCA_000414525_JCP8108         TDFQAAVEAAIKGVEIKPNTEFHRILQNTSVTAVILGGSANGAAKVVTGNIDTLKALIQEGANLSTSSPAVPIAYTT
GCA_002861905_UMB0830         TDFQAAVEAAIKGVEIKPNTEFHRILQNTSVTAVILGGSANGAAKVITGNVDTLKALIQEGANLSTSSPAVPIAYTT
GCA_003369895_N101            TDFQAAVEAAIKGVEIKPNTEFHRILQNTSVTAVILGGSANGAAKVITGNVDTLKALIQEGANLSTSSPAVPIAYTT
GCA_003369935_N153            TDFQAAVEAAIKGVEIKPNTEFHRILQNTSVTAVILGGSANGAAKVITGNVDTLKALIQEGANLSTSSPAVPIAYTT
GCA_003369965_N95             TDFQAAVEAAIKGVEIKPNTEFHRILQNTSVTAVILGGSANGAAKVITGNVDTLKALIQEGANLSTSSPAVPIAYTT
GCA_000414625_JCP7719         TDFQAAVEAAIKGVEIKPNTEFHRILQNTSVTAVILGGSANGAAKVITGNVDTLKALIQEGANLSTSSPAVPIAYTT
GCA_000414485_JCP8151B        TDFQAAVEAAIKGVEIKPNTEFHRILQNTSVTAVILGGSANGAAKVITGNVDTLKALIQEGANLSTSSPAVPIAYTT
GCA_000263635_00703Dmash      TDFQAAVEAAIKGVEIKPNTEFHRILQNTSVTAVILGGSANGAAKVITGNIDTLKALIQEGANLSTSSPAVPIAYTT
GCA_000165615_101             TDFQAAVEAAIKGVEIKPNTEFHRILQNTSVTAVILGGSANGAAKVITGNIDTLKALIQEGANLSTSSPAVPIAYTT
GCA_002884775_UMB1686         TDFQAAVEAAIKGVEIKPNTEFHRILQNTSVTAVILGGSANGAAKVITGNIDTLKALIQEGANLSTSSPAVPIAYTT
GCA_000263595_1500E           TDFQAAVEAAIKGVEIKPNTEFHRILQNTSVTAVILGGSANGAAKVITGNIDTLKALIQEGANLSTSSPAVPIAYTT
GCA_000414445_JCP8481B        TDFQAAVEAAIKGVEIKPNTEFHRILQNTSVTAVILGGSANGAAKVITGDINTLKSLIQEGANLSTSSPAVPIAYTT
GCA_000414465_JCP8481A        TDFQAAVEAAIKGVEIKPNTEFHRILQNTSVTAVILGGSANGAAKVITGDINTLKSLIQEGANLSTSSPAVPIAYTT
GCA_001660735_23-12           TDFQAAVEAAIKGVEIKPNTEFHRILQNTSVTAVILGGSANGAAKVITGNVDTLKALIQEGANLSTSSPAVPIAYTT
GCA_003408845_NR010           TDFQAAVEAAIKGVEIKPNTEFHRILQNTSVTAVILGGSANGAAKVVTGNIDTLKALIQEGANLSTSSPAVPIAYTT
GCA_003426445_NR037           TDFQAAVEAAIKGVEIKPNTEFHRILQNTSVTAVILGGSANGAAKVITGNIDTLKALIQEGANLSTSSPAVPIAYTT
GCA_002896555_KA00225         TDFQAAVEAAIKGVEIKPNTEFHRILQNTSVTAVILGGSANGAAKVITGNIDTLKALIQEGANMSTSSPAVPIAYTT
GCA_001546485_PSS_7772B       TDFQAAVEAAIKGVEIKPNTEFHRILQNTSVTAVILGGSANGAAKVITGDINTLKSLIQEGANLSTSSPAVPIAYTT
GCA_000025205_409-05          TDFQAAVEAAIKGVDIKPNTEFHRILQNTSVTAVILGGSANGAAKVITGNIDTLKALIQEGANLSTSSPAVPIAYTT
GCA_000176495_5-1             TDFQAAVEAAIKGVEIKPNTEFHRILQNTSVTAVILGGSANGAAKVITGNIDTLKALIQEGANLSTSSPAVPIAYTT
GCA_001953155_GV37            TDFQAAVEAAIKGVDIKPNTEFHRILQNTSVTAVILGGSANGAAKVITGNIDTLKALIQEGANLSTSSPAVPIAYTT
GCA_002884795_UMB1642         TDFQAAVEAAIKGVEIKPNTEFHRILQNTSVTAVILGGSANGAAKVITGNIDTLKALIQEGANLSTSSPAVPIAYTT
GCA_002884855_UMB0170         TDFQAAVEAAIKGVDIKPNTEFHRILQNTSVTAVILGGSANGAAKVITGNIDTLKALIQEGANLSTSSPAVPIAYTT
GCA_002884875_UMB0264         TDFQAAVEAAIKGVDIKPNTEFHRILQNTSVTAVILGGSANGAAKVITGNIDTLKALIQEGANLSTSSPAVPIAYTT
GCA_003397705_GS9838-1        TDFQAAVEAAIKGVEIKPNTEFHRILQNTSVTAVILGGSANGAAKVITGNIDTLKALIQEGANLSTSSPAVPIAYTT
GCA_003397745_GS10234         TDFQAAVEAAIKGVDIKPNTEFHRILQNTSVTAVILGGSANGAAKVITGNIDTLKALIQEGANLSTSSPAVPIAYTT
GCA_003408815_N72             TDFQAAVEAAIKGVEIKPNTEFHRILQNTSVTAVILGGSANGAAKVITGNIDTLKALIQEGANLSTSSPAVPIAYTT
GCA_002861125_UMB0912         TDFQAAVEAAIKGVEIKPNTEFHRILQNTSVTAVILGGSANGAAKVITGNIDTLKALIQEGANLSTSSPAVPIAYTT
GCA_002861145_UMB0913         TDFQAAVEAAIKGVEIKPNTEFHRILQNTSVTAVILGGSANGAAKVITGNIDTLKALIQEGANLSTSSPAVPIAYTT
GCA_003397635_UGent09.48      TDFQAAVEAAIKGVEIKPNTEFHRILQNTSVTAVILGGSANGAAKVITGNIDTLKALIQEGANLSTSSPAVPIAYTT
GCA_000263575_6420B           TDFQAAVEAAIKGVEIKPNTEFHRILQNTSVTAVILGGSANGAAKVITGNIDTLKALIQEGANLSTSSPAVPIAYTT
GCA_000176475_AMD             TDFQAAVEAAIKGVEIKPNTEFHRILQNTSVTAVILGGSANGAAKVITGNIDTLKALIQEGANLSTSSPAVPIAYTT
GCA_003293675_UGent06.41      TDFQAAVEAAIKGVEIKPNTEFHRILQNTSVTAVILGGSANGAAKVITGNIDTLKALIQEGANLSTSSPAVPIAYTT
GCA_002862065_UMB0682         TDFQAAVEAAIKGVEIKPNTEFHRILQNTSVTAVILGGSANGAAKVITGNIDTLKALIQEGANLSTSSPAVPIAYTT
GCA_001563665_CMW7778B        TDFQAAVEAAIKGVEIKPNTEFHRILQKTSVTAVILGGSADGAAKVITGNIDTLKALIQEGANLSTSSPAVPIAYTT
```

```
                                 380       390       400       410       420       430       440       450
WP_009567818.1_Sintermedius     SAQSPAVPISYTTSFVKDNSIATIQNNTDYIETKVTSYKDGALTLNHDGAFVARFYVYWEELGHDADGYETIRSRSW
WP_006730404.1_Liners           SFLKDNQVAVINNTADYIATKVTEYSNGKLILNHKGAYVARFFVDWDEVSYDSNGREVLTHHTWADNGRGRTSGFST
GCA_003397685_ATCC14018         SFVKDNEVATLQSNSDYIETKVSSYRNGYLTLDHRGAYVARYYIYWDEYGTEIDGTPYVRSRAWEGNGKYRTAHFNT
GCA_000159155_ATCC14019         SFVKDNEVATLQSNSDYIETKVSSYRNGYLTLDHRGAYVARYYIYWDEYGTEIDGTPYVRSRAWEGNGKYRTAHFNT
GCA_900637625_NCTC10287         SFVKDNEVATLQSNSDYIETKVSSYRNGYLTLDHRGAYVARYYIYWDEYGTEIDGTPYVRSRAWEGNGKYRTAHFNT
GCA_001042655_JCM11026          SFVKDNEVATLQSNSDYIETKVSSYRNGYLTLDHRGAYVARYYIYWDEYGTEIDGTPYVRSRAWEGNGKYRTAHFNT
GCA_900105405_DSM4944           SFVKDNEVATLQSNSDYIETKVSSYRNGYLTLDHRGAYVARYYIYWDEYGTEIDGTPYVRSRAWEGNGKYRTAHFNT
GCA_003034925_ATCC49145         SFVKDNEVATLQSNSDYIETKVSSYRNGYLTLDHRGAYVARYYIYWDEYGTEIDGTPYVRSRAWEGNGKYRTAHFNT
GCA_003812765_FDAARGOS_568      SFVKDNEVATLQSNSDYIETKVSSYRNGYLTLDHRGAYVARYYIYWDEYGTEIDGTPYVRSRAWEGNGKYRTAHFNT
GCA_002206225_FDAARGOS_296      SFVKDNEVATLQSNSDYIETKVSSYRNGYLTLDHRGAYVARYYIYWDEYGTEIDGTPYVRSRAWEGNGKYRTAHFNT
GCA_002861975_UMB0298           SFVKDNEVATLQSNSDYIETKVSSYRNGYLTLDHRGAYVARYYIYWDEYGTEIDGTPYVRSRAWEGNGKYRTAHFNT
GCA_002861965_UMB0386           SFVKDNEVATLQSNSDYIETKVSSYRNGYLTLDHRGAYVARYYIYWDEYGTEIDGTPYVRSRAWEGNGKYRTAHFNT
GCA_003397605_UGent25.49        SFVKDNEVATLQSNSDYIETKVSSYRNGYLTLDHRGAYVARYYIYWDEYGTEIDGTPYVRSRAWEGNGKYRTAHFNT
GCA_003408745_GH015             SFVKDNEVATLQSNSDYIETKVSSYRNGYLTLDHRGAYVARYYIYWDEYGTEIDGTPYVRSRAWEGNGKYRTAHFNT
GCA_002861945_UMB0770           SFVKDNEVATLQSNSDYIETKVSSYRNGYLTLDHRGAYVARYYIYWDEYGTEIDGTPYVRSRAWEGNGKYRTAHFNT
GCA_001049785_3549624           SFVKDNEVATLQSNSDYIETKVSSYRNGYLTLDHRGAYVARYYIYWDEYGTEIDGTPYVRSRAWEGNGKYRTAHFNT
GCA_000213955_HMP9231           SFVKDNEVATLQSNSDYIETKVSSYRNGYLTLDHRGAYVARYYIYWDEYGTEIDGTPYVRSRAWEGNGKYRTAHFNT
GCA_002861925_UMB0775           SFVKDNEVATLQSNSDYIETKVSSYRNGYLTLDHRGAYVARYYIYWDEYGTEIDGTPYVRSRAWEGNGKYRTAHFNT
GCA_003426545_NR001             SFVKDNEVATLQSNSDYIETKVSSYRNGYLTLDHRGAYVARYYIYWDEYGTEIDGTPYVRSRAWEGNGKYRTAHFNT
GCA_003585655_NR038             SFVKDNEVATLQSNSDYIETKVSSYRNGYLTLDHRGAYVARYYIYWDEYGTEIDGTPYVRSRAWEGNGKYRTAHFNT
GCA_002894105_DNF01149          SFVKDNEVATLQSNSDYIETKVSSYRNGYLTLDHRGAYVARYYIYWDEYGTEIDGTPYVRSRAWEGNGKYRTAHFNT
GCA_000263435_284V              SFVKDNEVATLQSNSDYIETKVSSYRNGYLTLDHRGAYVARYYIYWDEYGTEIDGTPYVRSRAWEGNGKYRTAHFNT
GCA_003426285_WP023             SFVKDNEVATLQSNSDYIETKVSSYRNGYLTLDHRGAYVARYYIYWDEYGTEIDGTPYVRSRAWEGNGKYRTAHFNT
GCA_002884835_UMB0768           SFVKDNEVATLQSNSDYIETKVSSYRNGYLTLDHRGAYVARYYIYWDEYGTEIDGTPYVRSRAWEGNGKYRTAHFNT
GCA_003397665_UGent09.07        SFVKDNEVATLQSNSDYIETKVSSYRNGYLTLDHRGAYVARYYIYWDEYGTEIDGTPYVRSRAWEGNGKYRTAHFNT
GCA_000263555_0288E             SFVKDNEVATLQSNSDYIETKVSSYRNGYLTLDHRGAYVARYYIYWDEYGTEIDGTPYVRSRAWEGNGKYRTAHFNT
GCA_000414685_JCP7276           SFVKDNEVATLQSNSDYIETKVSSYRNGYLTLDHRGAYVARYYIYWDEYGTEIDGTPYVRSRAWEGNGKYRTAHFNT
GCA_000414645_JCP7672           SFVKDNEVATLQSNSDYIETKVSSYRNGYLTLDHRGAYVARYYIYWDEYGTEIDGTPYVRSRAWEGNGKYRTAHFNT
GCA_003397755_UGent09.01        SFVKDNEVATLQSNSDYIETKVSSYRNGYLTLDHRGAYVARYYIYWDEYGTEIDGTPYVRSRAWEGNGKYRTAHFNT
GCA_000263515_00703C2mash       SFVKDNEVATLQSNSDYIETKVSSYRDGYLTLDHRGAYVARYYIYWDEYGTEIDGTPYVRSRAWEGNGKYRTSHFNT
GCA_000263615_00703Bmash        SFVKDNEVATLQTNSDYVETKVSSYRDGYLTLDHRGAYVARYYIYWDEYGTEIDGTPYVRSRAWEGNGKYRTAHFNT
GCA_003408835_N144              SFVKDNEVATLQSNSDYIETKVSSYRDGYLTLDHRGAYVARYYIYWDEYGTEIDGTPYVRSRAWEGNGKYRTSHFNT
GCA_001546445_GED7275B          SFVKDNEVATLQTNSDYVETKVSSYRDGYLTLDHRGAYVARYYIYWDEYGTEIDGTPYVRSRAWEGNGKYRTAHFNT
GCA_000414605_JCP8017A          SFVKDNEVATLQTNSDYVETKVSSYRDGYLTLDHRGAYVARYYIYWDEYGTEIDGTPYVRSRAWEGNGKYRTAHFNT
GCA_000414585_JCP8017B          SFVKDNEVATLQTNSDYVETKVSSYRDGYLTLDHRGAYVARYYIYWDEYGTEIDGTPYVRSRAWEGNGKYRTAHFNT
GCA_000414665_JCP7659           SFVKDNEVATLQSNSDYIETKVSSYRDGYLTLDHRGAYVARYYIYWDEYGTEIDGTPYVRSRAWEGNGKYRTSHFNT
GCA_003408775_N160              SFVKDNEVATLQTNSDYVETKVSSYRDGYLTLDHRGAYVARYYIYWDEYGTEIDGTPYVRSRAWEGNGKYRTAHFNT
GCA_000263655_6119V5            SFVKDNEVATLQTNSDYVETKVSSYRDGYLTLDHRGAYVARYYIYWDEYGTEIDGTPYVRSRAWEGNGKYRTAHFNT
GCA_000263535_75712             SFVKDNEVATLQTNSDYVETKVSSYRDGYLTLDHRGAYVARYYIYWDEYGTEIDGTPYVRSRAWEGNGKYRTAHFNT
GCA_003585755_NR039             SFVKDNEVATLQSNSDYIETKVSSYRNGYLTLDHRGAYVARYYIYWDEYGTEIDGTPYVRSRAWEGNGKYRTAHFNT
GCA_002862045_UMB0233           SFVKDNEVATLQTNSDYVGTKVSSYRDGYLTLDHRGAYVARYYIYWDEYGTEIDGTPYVRSRAWEGNGKYRTAHFNT
GCA_000214315_315-A             SFVKDNEVATLQTNSDYVETKVSSYRDGYLTLDHRGAYVARYYIYWDEYGTEIDGTPYVRSRAWEGNGKYRTAHFNT
GCA_002862005_UMB0032B          SFVKDNEVATLQTNSDYVETKVSSYRDGYLTLDHRGAYVARYYIYWDEYGTEIDGTPYVRSRAWEGNGKYRTAHFNT
GCA_002862015_UMB0032A          SFVKDNEVATLQTNSDYVETKVSSYRDGYLTLDHRGAYVARYYIYWDEYGTEIDGTPYVRSRAWEGNGKYRTAHFNT
GCA_002861165_UMB0061           SFVKDNEVATLQSNSDYIETKVSSYRNGYLTLDHRGAYVARYYIYWDEYGTEIDGTPYVRSRAWEGNGKYRTAHFNT
GCA_001660755_18-4              SFVKDNEVATLQSNSDYIETKVSSYRNGYLTLDHRGAYVARYYIYWDEYGTEIDGTPYVRSRAWEGNGKYRTAHFNT
GCA_000165635_41V               SFVKDNEVATLQTNSDYVETKVSSYRNGYLTLDHRGAYVARYYIYWDEYGTEIDGTPYVRSRAWEGNGKYRTAHFNT
GCA_000263475_55152             SFVKDNEVATLQTNSDYVETKVSSYRNGYLTLDHRGAYVARYYIYWDEYGTEIDGTPYVRSRAWEGNGKYRTAHFNT
GCA_000263495_1400E             SFVKDNEVATLQTNSDYVETKVSSYRNGYLTLDHRGAYVARYYIYWDEYGTEIDGTPYVRSRAWEGNGKYRTAHFNT
GCA_000414525_JCP8108           SFVKDNEVATLQTNSDYVETKVSSYRNGYLTLDHRGAYVARYYIYWDEYGTEIDGTPYVRSRAWEGNGKYRTAHFNT
GCA_002861905_UMB0830           SFVKDNEVATLQTNSDYVETKVSSYRDGYLTLDHRGAYVARYYIYWDEYGTEIDGTPYVRSRAWEGNGKYRTAHFNT
GCA_003369895_N101              SFVKDNEVATLQTNSDYVETKVSSYRDGYLTLDHRGAYVARYYIYWDEYGTEIDGTPYVRSRAWEGNGKYRTAHFNT
GCA_003369935_N153              SFVKDNEVATLQTNSDYVETKVSSYRDGYLTLDHRGAYVARYYIYWDEYGTEIDGTPYVRSRAWEGNGKYRTAHFNT
GCA_003369965_N95               SFVKDNEVATLQTNSDYVETKVSSYRDGYLTLDHRGAYVARYYIYWDEYGTEIDGTPYVRSRAWEGNGKYRTAHFNT
GCA_000414625_JCP7719           SFVKDNEVATLQTNSDYVETKVSSYRDGYLTLDHRGAYVARYYIYWDEYGTEIDGTPYVRSRAWEGNGKYRTAHFNT
GCA_000414485_JCP8151B          SFVKDNEVATLQTNSDYVETKVSSYRDGYLTLDHRGAYVARYYIYWDEYGTEIDGTPYVRSRAWEGNGKYRTAHFNT
GCA_000263635_00703Dmash        SFVKDNEVATLQTNSDYVETKVSSYRDGYLTLDHRGAYVARYYIYWDEYGTEIDGTPYVRSRAWEGNGKYRTAHFNT
GCA_000165615_101               SFVKDNEVATLQTNSDYVETKVSSYRDGYLTLDHRGAYVARYYIYWDEYGTEIDGTPYVRSRAWEGNGKYRTAHFNT
GCA_002884775_UMB1686           SFVKDNEVATLQTNSDYVETKVSSYRDGYLTLDHRGAYVARYYIYWDEYGTEIDGTPYVRSRAWEGNGKYRTAHFNT
GCA_000263595_1500E             SFVKDNEVATLQTNSDYVETKVSSYRDGYLTLDHRGAYVARYYIYWDEYGTEIDGTPYVRSRAWEGNGKYRTAHFNT
GCA_000414445_JCP8481B          SFVKDNEVATLQSNSDYIETKVSSYRDGYLTLDHRGAYVARYYIYWDEYGTEIDGTPYVRSRAWEGNGKYRTSHFNT
GCA_000414465_JCP8481A          SFVKDNEVATLQSNSDYIETKVSSYRDGYLTLDHRGAYVARYYIYWDEYGTEIDGTPYVRSRAWEGNGKYRTSHFNT
GCA_001660735_23-12             SFVKDNEVATLQTNSDYVGTKVSSYRDGYLTLDHRGAYVARYYIYWDEYGTEIDGTPYVRSRAWEGNGKYRTAHFNT
GCA_003408845_NR010             SFVKDNEVATLQTNSDYVETKVSSYRDGYLTLDHRGAYVARYYIYWDEYGTEIDGTPYVRSRAWEGNGKYRTAHFNT
GCA_003426445_NR037             SFVKDNEVATLQSNSDYIETKVSSYRNGYLTLDHRGAYVARYYIYWDEYGTEIDGTPYVRSRAWEGNGKYRTAHFNT
GCA_002896555_KA00225           SFVKDNEVATLQSNSDYIETKVSSYRDGYLTLDHRGAYVARYYIYWDEYGTEIDGTPYVRSRAWEGNGKYRTAHFNT
GCA_001546485_PSS_7772B         SFVKDNEVATLQSNSDYIETKVSSYRDGYLTLDHRGAYVARYYIYWDEYGTEIDGTPYVRSRAWEGNGKYRTSHFNT
GCA_000025205_409-05            SFVKDNEVATLQSNSDYVETKVSAYRDGYLTLDHRGAYVARYYVYWDEYGTDIDGTPYVRSRAWEGNGKYRTAHFST
GCA_000176495_5-1               SFVKDNEVATLQSNSDYVETKVSAYRDGYLTLDHRGAYVARYYVYWDEYGTDIDGTPYVRSRAWEGNGKYRTAHFST
GCA_001953155_GV37              SFVKDNEVATLQSNSDYVETKVSAYRDGYLTLDHRGAYVARYYVYWDEYGTDIDGTPYVRSRAWEGNGKYRTAHFST
GCA_002884795_UMB1642           SFVKDNEVATLQSNSDYVETKVSAYRDGYLTLDHRGAYVARYYVYWDEYGTDIDGTPYVRSRAWEGNGKYRTAHFST
GCA_002884855_UMB0170           SFVKDNEVATLQSNSDYVETKVSAYRDGYLTLDHRGAYVARYYVYWDEYGTDIDGTPYVRSRAWEGNGKYRTAHFST
GCA_002884875_UMB0264           SFVKDNEVATLQSNSDYVETKVSAYRDGYLTLDHRGAYVARYYVYWDEYGTDIDGTPYVRSRAWEGNGKYRTAHFST
GCA_003397705_GS9838-1          SFVKDNEVATLQSNSDYVETKVSAYRDGYLTLDHRGAYVARYYVYWDEYGTDIDGTPYVRSRAWEGNGKYRTAHFST
GCA_003397745_GS10234           SFVKDNEVATLQSNSDYVETKVSAYRDGYLTLDHRGAYVARYYVYWDEYGTDIDGTPYVRSRAWEGNGKYRTAHFST
GCA_003408815_N72               SFVKDNEVATLQSNSDYVETKVSAYRDGYLTLDHRGAYVARYYVYWDEYGTDIDGTPYVRSRAWEGNGKYRTAHFST
GCA_002861125_UMB0912           SFVKDNEVATLQSNSDYVETKVSAYRDGYLTLDHRGAYVARYYVYWDEYGTDIDGTPYVRSRAWEGNGKYRTAHFST
GCA_002861145_UMB0913           SFVKDNEVATLQSNSDYVETKVSAYRDGYLTLDHRGAYVARYYVYWDEYGTDIDGTPYVRSRAWEGNGKYRTAHFST
GCA_003397635_UGent09.48        SFVKDNEVATLQSNSDYVETKVSAYRDGYLTLDHRGAYVARYYVYWDEYGTDIDGTPYVRSRAWEGNGKYRTAHFST
GCA_000263575_6420B             SFVKDNEVATLQSNSDYVETKVSAYRDGYLTLDHRGAYVARYYVYWDEYGTDIDGTPYVRSRAWEGNGKYRTAHFST
GCA_000176475_AMD               SFVKDNEVATLQSNSDYVETKVSAYRDGYLTLDHRGAYVARYYVYWDEYGTDIDGTPYVRSRAWEGNGKYRTAHFST
GCA_003293675_UGent06.41        SFVKDNEVATLQSNSDYVETKVSAYRDGYLTLDHRGAYVARYYVYWDEYGTDIDGTPYVRSRAWEGNGKYRTAHFST
GCA_002862065_UMB0682           SFVKDNEVATLQSNSDYVETKVSAYRDGYLTLDHRGAYVARYYVYWDEYGTDIDGTPYVRSRAWEGNGKYRTAHFST
GCA_001563665_CMW7778B          SFVKDNEVATLQSNSDYIETKVSSYRDGYLTLDHRGAYVARYYIYWDEYGTEVDGTPYVRSRAWEGNGKYRTAHFNT
```

Supplementary Figure S1: Alignment of the first 455 residues of intermedilysin, inerolysin, and vaginolysin amino acid sequences. Solid red boxes are residues conserved throughout all toxins. Open black boxes with residues in red font are conserved among all VLY sequences. Blue boxes denote transmembrane helices. Yellow boxes (residues 423-440) denote residues implicated in binding to CD59 by x-ray crystallography (Lawrence, S.L.; Gorman, M.A.; Feil, S.C.; Mulhern, T.D.; Kuiper, M.J.; Ratner, A.J.; Tweten, R.K.; Morton, C.J.; Parker, M.W. Structural Basis for Receptor Recognition by the Human CD59-Responsive Cholesterol-Dependent Cytolysins. Struct. Lond. Engl. 1993 2016, 24, 1488–1498).

Figure S2.Alignment of VLY undecapeptide and CD59 regions reveals distinct VLY

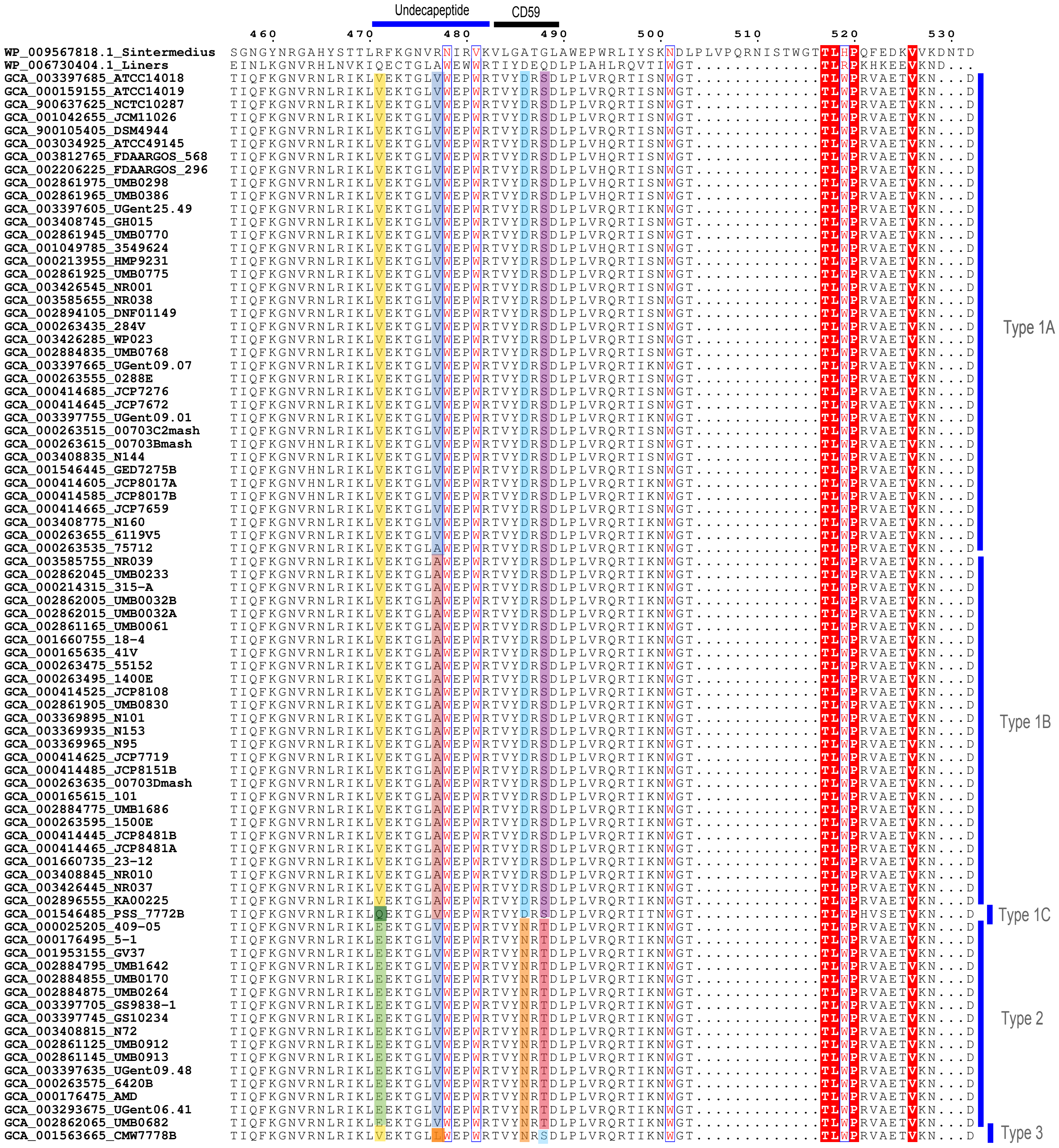

Figure S2. Alignment of VLY undecapeptide and CD59 regions (Hughes, T.R.; Ross, K.S.; Cowan, G.J.M.; Sivasankar, B.; Harris, C.L.; Mitchell, T.J.; Morgan, B.P. Identification of the high affinity binding site in the Streptococcus intermedius toxin intermedilysin for its membrane receptor, the human complement regulator CD59. Mol. Immunol. 2009, 46, 1561–1567) reveals distinct VLY types. The alignment was generated using the undecapeptide and CD59 regions of VLY amino acid sequences extracted from 84 Gardnerella spp. genome sequences available on NCBI.

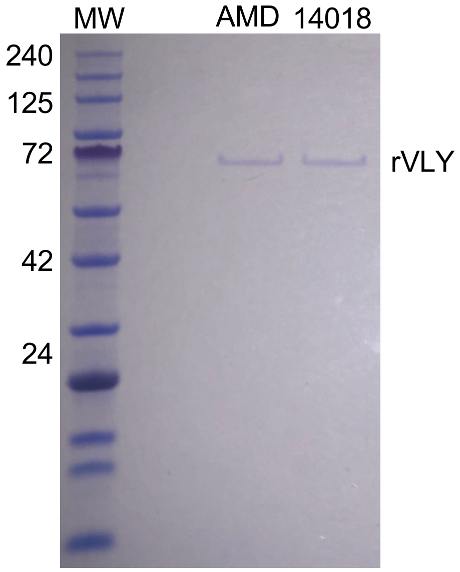

Figure S3. Equivalent purity and concentration of Type 1 and Type 2 rVLY. SDS-PAGE gel on which 100 ng rVLY from *G. leopoldii* AMD (type 2) and *G. vaginalis* ATCC 14018 (type 1A) were loaded. The two toxin preparations appear to contain equal concentrations of 6x-His tagged VLY and few contaminants. The tagged recombinant protein is ~73kDa. MW is the BLUEstain protein Ladder (GoldBio).

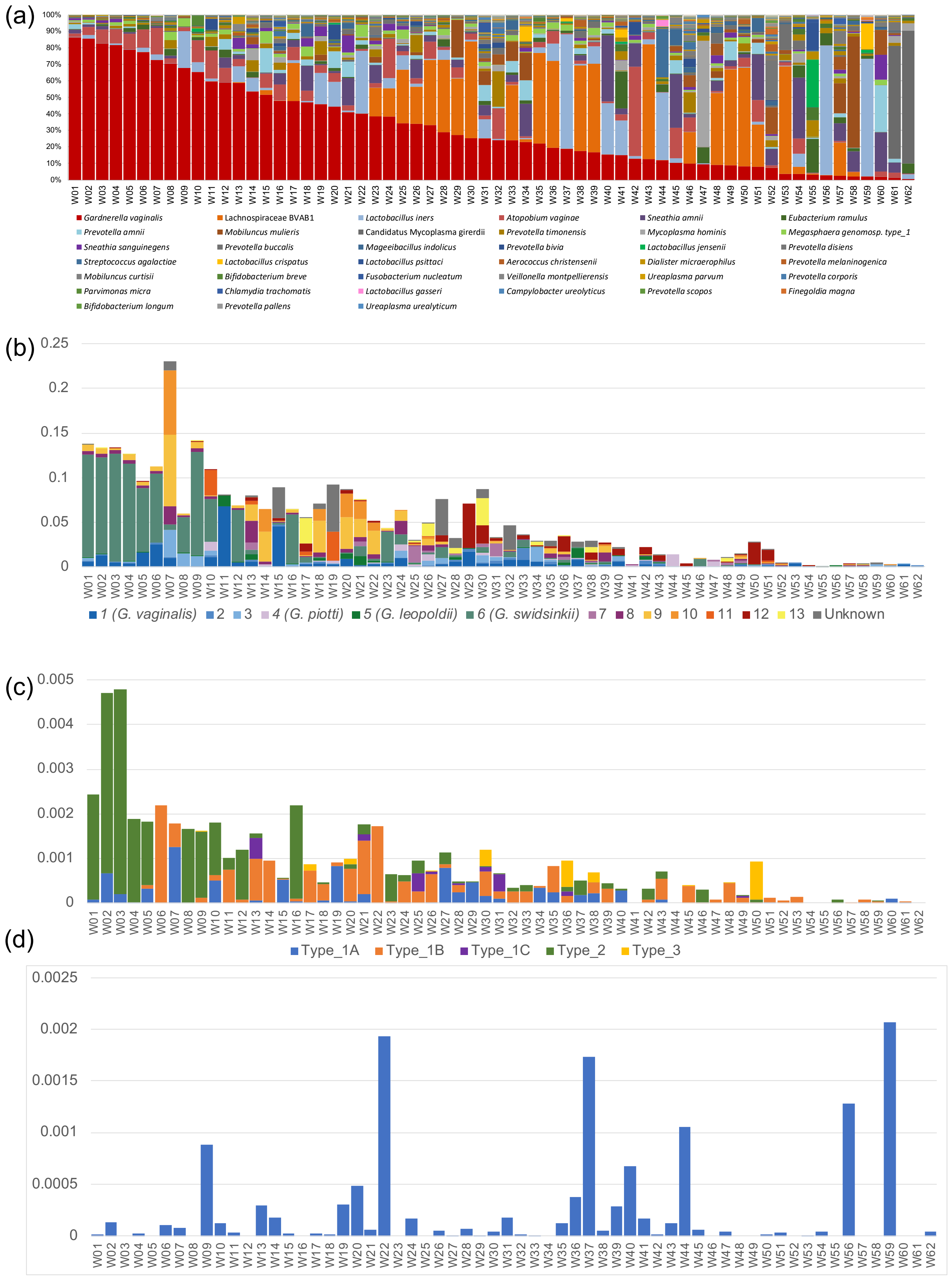

Supplementary Figure S4. (a) Relative abundances of bacterial species in vaginal microbiomes based on whole metagenome shotgun (WMGS) sequence profiles was determined using ClarkS classifier. **(b)** The abundance of *Gardnerella* genomic species was determined using cpn60 reads from the WMGS data (the data is normalized by the total number of non-human reads). The abundance of **(c)** vly types and **(d)** inerolysin based on WMGS data (the data is normalized by the total number of non-human reads).
